# Investigating the Role of Neutrophil Extracellular Traps as a Therapeutic Target in Traumatic Brain Injury: a Systematic Review and Meta-analysis

**DOI:** 10.1007/s12035-025-05053-7

**Published:** 2025-05-23

**Authors:** Elliott Slough, Anna Pitt-Francis, Antonio Belli, Zubair Ahmed, Valentina Di Pietro, Andrew R. Stevens

**Affiliations:** 1 Neuroscience and Ophthalmology, Department of Inflammation and Ageing, School of Infection, Inflammation and Immunology, University of Birmingham, Edgbaston, Birmingham, UK; 2 Department of Neurosurgery, Queen Elizabeth Hospital Birmingham, Edgbaston, Birmingham, UK; 3 University Hospitals Birmingham NHS Trust, Edgbaston, Birmingham, UK; 4 Centre for Trauma Sciences Research, University of Birmingham, Edgbaston, Birmingham, UK; 5 Neuroscience and Ophthalmology Group, Aitken Institute for Clinical Research, University of Birmingham, Edgbaston Birmingham, Robert, B15 2 TT UK

**Keywords:** Neuroinflammation, Neutrophils, Neutrophil extracellular traps (NETs), Traumatic brain injury (TBI), Animal models, NET modulation

## Abstract

Traumatic brain injury (TBI) is a leading cause of morbidity and mortality worldwide, with few treatment options to mitigate secondary injury. Neutrophil extracellular traps (NETs) may present a potential target for therapy. The systematic review objectives were to characterise NET formation as a feature following TBI; assess the effect of NET modulatory strategies on outcomes; and investigate the relative efficacy of NET modulatory methods. A systematic review was performed, with inclusion criteria of pre-clinical or clinical studies using any model or severity of TBI, and any investigation of the pathophysiological role of NETs and/or modulation of NETs for therapeutic benefit. Following search completion, 849 records were identified with 13 studies eligible for inclusion. All 13 studies characterised NET formation in blood and brain tissue from pre-clinical TBI models, whilst four studies also demonstrated NET formation in serum and brain tissue of TBI patients. Meta-analysis (where ≥ 3 studies reported outcomes) identified that NET modulation was associated with significant improvement of outcomes in preclinical studies, in both modified neurological severity score and latency to falls. No difference in efficacy was identified between NET modulatory methods after sub-group analysis. In addition, the overall risk of bias was judged as high in the included studies. This systematic review and meta-analysis demonstrated that NETs present a promising TBI therapeutic target for future clinical validation. However, the high bias limits this systematic review, and further high-quality studies are required to make definitive conclusions about NET utility as a viable therapeutic strategy in TBI.

**Supplementary Information:**

The online version contains supplementary material available at 10.1007/s12035-025-05053-7.

## Background

Traumatic brain injury (TBI) is a leading cause of mortality and morbidity worldwide [1]. The neurological damage caused by TBI is understood as two discrete phases: primary injury, sustained during the initial trauma; and secondary injury, which develops over time and involves a series of molecular cascades set in motion by the primary injury. Secondary injury cascades result in further damage through apoptosis, necrosis, blood–brain barrier (BBB) dysfunction, and neuroinflammation, which cause further neurological injury [2, 3]. Improved scientific understanding of these mechanisms is important for the identification of novel therapeutic targets for mitigating secondary damage and improving functional outcomes after injury [4].

Following TBI, neuroinflammation and neuronal injury are fundamental in secondary injury pathogenesis, leading to motor and cognitive deficits [5, 6]. Rapid increases in the number and brain infiltration of neutrophils have been demonstrated immediately after and in the days following TBI [7–10] and are proposed as a major contributor to inflammation-induced secondary brain injury [11]. However, inflammation and neutrophils also play critical roles in preserving neural tissue, promoting regeneration, and aiding recovery following brain injury [12].

In 2004, Brinkmann and colleagues discovered that neutrophils can extrude an extracellular mesh structure composed of cytosolic and granular proteins scaffolded on citrullinated histones and decondensed chromatin, termed ‘neutrophil extracellular traps’ (NETs) [13]. The role of NETs was originally thought to exclusively consist of trapping and immobilising cells and pathogens [14]. However, NET release has been discovered to be a highly variable phenomenon with differing types of NETs produced dependent on context [15]. NETs are induced by a variety of stimuli and have the capacity to actively participate in multiple cellular and molecular cascades through the release of various component mediators [16]. NETs have subsequently been implicated in multiple central nervous system pathologies [15] and in non-infectious trauma [14].

Importantly, NET generation has been evidenced in blood samples from patients during the acute response to sterile traumatic injury [17]. NETs have also been identified in the brain parenchyma of ischaemic stroke patients, with elevated plasma NETs in this group correlating with worse outcomes [18]. In another instance of acute brain injury, NET presence was detected in brain tissues of spontaneous intracerebral haemorrhage patients, with NETs shown to infiltrate haematoma injury areas [19]. Additionally, within the context of subarachnoid haemorrhage, increased NET formation correlated with severity within this patient population and formation peaked at 24 h in the brain after subarachnoid haemorrhage remodelling [20]. NET formation aggravated neuronal damage and brain oedema, as well as mediating neuroinflammation by promoting microglial activation, with inhibition of NETs resulting in a reduction of these deficits.

Targeting and reducing NETs may therefore provide an innovative selective immunomodulatory strategy within the context of TBI that maintains the benefit of an immune response while ameliorating pernicious secondary brain injuries and neurological deficits. There is a requirement for establishing an evidence base regarding NETs as a viable target for therapy in the context of TBI secondary injury. This systematic review and meta-analysis aimed to define the current evidence for the utility of NETs as a target for therapy, elucidating a role for NET modulation, guiding future study and clinical direction. The specific aims of this systematic review are to [1] characterise the evidence for NET formation after TBI, and correlation with outcome; [2] assess the effects of NET modulation on functional outcomes and neurological deficits following TBI; and [3] explore the relative efficacy of NET modulatory methods for improving outcomes after TBI.

## Main Text

### Methods

A systematic review of the literature was performed on 1 st July 2024 and was conducted in accordance with the methodology of the Cochrane Handbook for Systematic Reviewers [21] and is presented in accordance with the Preferred Reporting Items for Systematic Reviews and Meta-analyses (PRISMA) guidelines. In accordance with the guidelines, the systematic review protocol was registered with the International Prospective Register of Systematic Reviews [22].

### Literature Search

Searches were performed by two reviewers (ES, APF) independently and the following databases were searched from inception to 1 st July 2024: PUBMED; Elsevier (Science Direct); Cochrane Trials Tab; Clinical Trials.gov; Embase; Scopus; Web of Science; and Google Scholar. Reference lists from key articles and articles identified for inclusion were manually searched ensuring selection was fully encompassing of relevant articles. No limitations were imposed on searches. The following is an example of the search strategy implemented (for PUBMED):

((NETS) OR (Neutrophil Extracellular traps)) AND ((TBI) OR (Traumatic Brain Injury) OR (diffuse axonal injury) OR (closed brain injury) OR (head injury)).

### Study Selection

All titles and abstracts were screened independently by the two reviewers (ES, APF) against the inclusion criteria, followed by full text screening. Inclusion criteria were as follows: pre-clinical studies of TBI (using any model or severity) or clinical studies including patients with TBI (of any severity); any investigation of the pathophysiological role of NETs, and/or modulation of NETs for therapeutic benefit; and any study design or methodology. Exclusion criteria were: studies examining other types of non-neutrophil nets (including perineural nets); pre-prints; studies that have not been subjected to peer review; and unpublished data. Reasons for excluding studies were recorded.

### Data Extraction

Data were extracted from included studies using a pre-designed form and collated into a matrix. For preclinical studies, recorded information included: subject characteristics (species type; age; sex; weight); injury model and severity; comparator; pathophysiological process investigated; and experimental design. Where pre-clinical models investigated the presence of NETs, outcomes and outcome measures were extracted. Where pre-clinical models investigated NET modulation, data regarding NET modulatory therapy variables were extracted (NET modulatory strategy used, administration method, dosing strategy), alongside comparators and relevant outcomes. Findings of any mechanistic sub-studies were also recorded. Regarding clinical studies, in the context of demonstrating NET presence, data extracted included: patient injury and *n* number, injury severity, specimen obtained, comparator and *n* number, outcomes, and outcomes measures. Any other information deemed relevant was also recorded in a section marked ‘other’. Where insufficient data was available, authors were contacted for further detail. When values were not reported directly and not available after contacting the corresponding authors, data was carefully extrapolated from published figures. For the meta-analysis, data was extracted regarding modified neurological severity score (mNSS) and latency to falls (s) on the rotarod test. Findings regarding other functional outcomes and TBI-associated deficits were extracted for narrative presentation.

### Synthesis of Results

A combination of meta-analysis and narrative synthesis is used to present available data, with information presented in texts, tables and figures explaining the findings of the studies included. When outcome measures were sufficiently homogenous and where outcomes were reported in n ≥ 3 studies, a meta-analysis was conducted and presented accordingly. Where outcomes were reported at a range of timepoints, endpoint outcomes were used in a standardised mean difference model. When multiple treatment groups were compared with the same control group, the combined average of the means and standard deviations (SDs) of the treatment groups were calculated and compared with control to avoid introducing bias by comparisons of multiple treatment groups to one control group. Outcomes from included studies which were not suitable for meta-analysis are presented in narrative and tabular form.

### Statistical Analysis

Review Manager 5.4 [23] was used for meta-analysis and graphical presentation. Mean, SD, and *n* number were extracted for the meta-analysis. SD was calculated using standard error of the mean (SEM) and *n* number, where SEM was presented. Standardised mean difference models were used due to heterogenous data reporting to calculate effect size with a 95% confidence interval (CI). Fixed effect models were used as heterogeneity was calculated by Chi squared statistics as ≤ 50% [24].

### Risk of Bias Assessment

The risk of bias (RoB) for included animal studies was assessed using the SYRCLE RoB tool [25] to assess methodological quality. For each individual study, the procedures undertaken regarding each domain in the tool was described and a judgement of low, high, or unclear RoB was assigned to each domain. When the RoB was unable to be determined, due to for example insufficiencies in manuscript information, the RoB was graded unclear. For human studies, the RoB was assessed using ROBINS-E tool for assessing exposures [26], adapting this tool for use assessing elements where NET presence in patients where investigated. A judgement of low, some concerns or high bias was assigned for each domain, with overall RoB determined as low, fair, or high for each study included. This was completed by two reviewers, independently in duplicate. Discrepancies were discussed with a third party (AS) and a resolution agreed on review.

## Results

### Study Selection

**Fig. 1:**
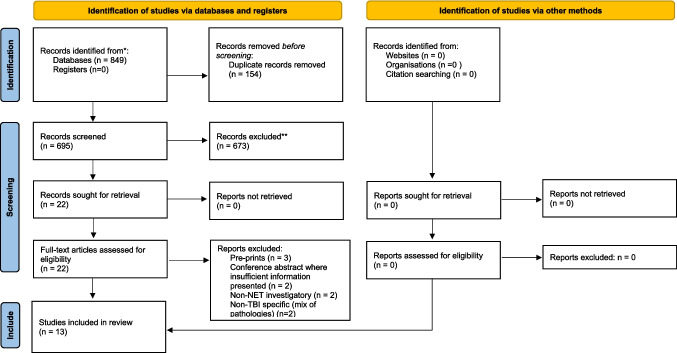
PRISMA 2020 flow diagram for new systematic reviews which included searches of databases, registers, and other sources

The systematic search identified 849 records with 695 after removal of duplicates. After title and abstract screening, 22 full texts articles were obtained for eligibility assessment. Thirteen studies met the inclusion/exclusion criteria, with use of pre-clinical in vivo models described in all 13 studies [27–39]. Five of the studies also described data from human samples [27, 29, 33, 34, 38]. No solely human study of NETs in TBI were identified. The search strategy and results are detailed in the PRISMA flow chart (Fig. 1).

### Study Characteristics

Each of the 13 studies included in the review had a pre-clinical TBI model on which the papers were primarily based (Table 1) [27–39]. Five studies used adult Sprague–Dawley rats [28, 30, 31, 36, 39], seven used C57BL/6 adult mice [27, 29, 32–35, 37] and one used CD-1, C3H/OuJ and C3HHeJ adult mice [38]. Seven studies utilised controlled cortical impact (CCI) as the injury model [27, 31–33, 35, 37, 38]; two used weight drop [29, 34]; one used a lateral fluid percussion injury model [28]; and two used a mechanical closed injury model to create a diffuse axonal injury (DAI). DAI was administered through lateral translation combined with rotation in coronal plane to induce the injury and realise the angular/linear acceleration and deceleration forces required [36, 39]. One study which implemented a surgical brain injury model was also included as it is effectively a traumatic model but differs in that there is a partial resection rather than impact [30].

**Table 1:** Summary table of included studies and their characteristics

Study ID	Species	Species characteristics	TBI model	Severity	Comparator	Pathophysiological process investigated
Summary	All studies included in the review consisted of pre-clinical rodent models	The animals used in models were primarily adult males of standard age and weights	CCI and mechanical closed injury model for DAI were common models used	Severity ranged from mild to severe, with severe models were used in the majority of cases	All but one study (in which no information was provided) used sham injured controls	A wide variety of pathophysiological processes were investigated by included studies
Cao et al., 2023	C57BL/6 mice	Adult (8–10 weeks); male; 25–30 g	CCI	Severe	TBI sham	Neuronal pyroptosis; neuronal ER-stress; neuroinflammation; sympathetic hyperactivity
Gu et al., 2021	Sprague Dawley rats	Age n/s; male; 220–250 g	Lateral fluid percussion injury	Severe	TBI sham	TBI-induced ALI; cerebral oedema; neuronal degeneration; sympathetic hyperactivity
Jin et al., 2023	C57BL/6 mice	Adult (12–16 weeks); male; 22–25 g	Closed-head injury using weight drop device	Severe	TBI sham	Coagulopathy; endothelial dysfunction; BBB dysfunction; cerebral oedema;
Li et al., 2024	Sprague Dawley rats	Adult (8–9 weeks); male; 270–320 g	SBI *	Severe	SBI sham	Neuroinflammation, cerebral oedema, neuronal apoptosis
Liu et al., 2012	Sprague Dawley rats	Adult rat; sex n/s; weight n/s	CCI	Not specified	N/A	N/A **
Liu et al., 2022	C57BL/6 mice	Adult (6–8 weeks); mixed sex;	CCI	Moderate	TBI sham	BBB dysfunction
Mi et al., 2023	C57BL/6 mice	Adult (8–10 weeks); male; 20–25 g	CCI	Severe	TBI sham	Neuronal ER-stress; neuronal apoptosis
Mu et al., 2024	C57BL/6 mice	Adult; 6–8 weeks; male; 20–25 g	Beating device weight drop	Mild/moderate	TBI sham	Neurovascular injury; cerebral oedema; BBB dysfunction; neuronal apoptosis; neuroinflammation
Pedragosa et al., 2021	C57BL/6 mice	Adult (8–10 weeks); male; 25–30 g	CCI	Moderate	N/A	N/A **
Qu et al., 2023	Sprague Dawley rats	Adult (8 weeks); male; 230–250 g	Mechanical closed injury model for DAI	Severe	DAI sham	Sympathetic hyperactivity; neuroinflammation
Shi et al., 2023	C57BL/6 mice	Adult (8–10 weeks); male; 25–30 g	CCI	Severe	TBI sham	Cerebral oedema; tissue loss; neuroinflammation; neurovascular injury; BBB dysfunction; neuronal apoptosis; neuronal ER-stress; sympathetic hyperactivity
Vaibhav et al., 2020	CD-1, C3H/OuJ, C3H/HeJ mice	Adult; mixed sex; weight n/s	CCI	Severe	TBI sham	Neurovascular injury; cerebral oedema
Zhu et al., 2021	Sprague Dawley rats	Adult; male; 230–280 g	Mechanical closed injury model for DAI	Severe	DAI sham	Sympathetic hyperactivity

*ALI* acute lung injury, *BBB* blood–brain barrier, *CCI*  controlled cortical impact, *DAI*  diffuse axonal injury, *ER-stress*  Endoplasmic-reticulum stress, *N/A*  not applicable, *SBI*  surgical brain injury, *TBI* traumatic brain injury.

*Partial right frontal lobectomy with two incisions: 2 mm lateral to the sagittal suture and 1 mm proximal to the coronal suture, with depth extending to the skull base

** No pathophysiological process investigated in regard to effect of NETs

Injuries were classified (based on manuscript information including severity stated, neurological function evaluations and method of injury induction) as severe in nine studies [27–30, 33, 36–39], moderate in two studies [32, 35], and mild/moderate in one study [34]. The severity of TBI in one study was indeterminable as insufficient information was reported [31]. All studies identified used in vivo models of TBI.

Within the 13 studies identified, five contained clinical elements investigating neutrophils and NETs in relation to TBI in human participants [27, 29, 33, 34, 38]. Four studies investigated NET formation in TBI patients with severities of moderate to severe, while one investigated the correlation between neutrophil count and severity of brain injuries, in mild to severe injuries. Samples collected were primarily blood, with one study also collecting TBI patients’ brain tissue specimens. Further detail can be found in the section regarding NET presence in humans.

### NET Formation Post-TBI

#### NET Presence in Pre-clinical Models

All studies identified NET presence following TBI in pre-clinical models [27–39] (Table 2), with NET components citrullinated histone H3 (Cit-H3), myeloperoxidase (MPO), and neutrophil elastase (NE) as the predominant markers used to detect and quantify NET presence. Techniques primarily utilised immunofluorescence (quantification and imaging, with co-localisation of markers) and Western blot. In all studies, there was a statistically significant increase in NET markers, including in blood [29, 30, 32, 34, 37–39], lungs [28] and brain tissues [27, 29–39], in samples from TBI animal models compared to sham-injured controls. Extracellular structures with NET-like morphologies and NET component markers were also observed by microscopy in TBI animals compared to sham-injured controls [30, 38]. NET generation increased in a time-dependent manner, with a trend in peaking at around 72 h [30, 33, 36, 37], and persisting for up to 14 days [37].

**Table 2:** NET presence in pre-clinical TBI models

Study ID	NET presence significantly demonstrated (*p* < 0.05)	Method used to evidence NETs (*n* = number, where available)	NET locality	NET markers	Temporal distribution of NETs post injury
Summary	All studies (*n* = 13) were successful in significantly demonstrating the presence of NETs in a variety of TBI models compared to sham-injured controls. The presence of NETs was observed in the blood and various brain regions of injured animals, through a range of NET markers, with levels peaking around 72 h and persisting for up to 14 days. Protein quantification methods such as western blotting and immunofluorescence were commonly employed to demonstrate the presence of NETs				
Cao et al., 2023	Yes	Western blot (*n* = 6) Immunofluorescence	Peri-contused cortex Brain tissue	MPO, PAD4, Cit-H3 Cit-H3, Ly6G	Elevated at Day 3 Elevated at Day 3
Gu et al., 2021	Yes	Western blot (*n* = 6)	Lung tissue	MPO, NE, Cit-H3	Elevated from 5 min to end point measurement (12 h)
Jin et al., 2023	Yes	ELISA (*n* = 18) Immunofluorescence	Blood Brain tissue	Cit-H3 Cit-H3, Ly6G	Elevated at 3 h and 6 h (endpoint measurement) N/S
Li et al., 2024	Yes	ELISA (n = 6) Western blot (n = 6) SEM	Blood Brain tissue Peri-contused brain tissue	Cit-H3, MPO Cit-H3 NET-like structures	Elevated at 12 h, 1-day, 3-day, 7-day; peaks at day 1 Elevated at 12 h, 1-day, 3-day, 7-day; peaks at day 3 Imaged at 3 days
Liu et al., 2012	Yes	Histology and assays	Traumatised area	MPO, NE, Neutrophil accumulation	Elevated at 12 h, 24 h, 72 h
Liu et al., 2022	Yes *	Immunofluorescence FACS	Blood Brain tissue	Cit-H3, MPO Cit-H3, MPO	Elevated at 24 h N/S
Mi et al., 2023	Yes	Western blot (n = 6) Immunofluorescence (n = 5)	Ipsilateral cortex Peri-contused cortex	PAD4 PAD4, MPO	Elevated at 6 h, 12 h, 24 h, 72 h, 5-day, 7-day; peaks at 72 h Elevated at 24 h and 72 h; peaks at 72 h
Mu et al., 2024	Yes	Flow cytometry Immunofluorescence	Blood Brain tissue	Cit-H3, Ly6G Cit-H3, MPO	Elevated at day 3 Elevated at day 3
Pedragosa et al., 2021	Yes **	Immunofluorescence	Brain tissue	Cit-H3	Elevated at day 1 and day 4
Qu et al., 2023	Yes	Western blotting (n = 3) Immunofluorescence	PVN PVN	Cit-H3, HMGB1 MPO, Cit-H3,	Elevated at day 1, day 2, day 3, day 5, day 7; peaks at 3 days N/S
Shi et al., 2023	Yes	Western blotting (n = 6) ELISA (n = 12) Immunofluorescence (n = 6)	Ipsilateral cortex Blood; cortical areas Contused cortex	Cit-H3, PAD4 MPO Cit-H3, MPO; PAD4, MPO	Elevated at 6 h and persists up to 14 days (Cit-H3), elevated at 1 day and persist up to 5 days (PAD4); both peaks at day 3 Elevated at day 1 and increased elevation at day 3 Elevated at day 1 and increased elevation at day 3
Vaibhav et al., 2020	Yes	Flow cytometry (n = 5) SEM; immunogold labelling (n = 5)	Blood; brain tissue Peri-contused cortex; peri-contused vasculature	Cit-H3, NE, MPO NET-like structures; Cit-H3, NE	Elevated at 24 h Imaged at 24 h
Zhu et al., 2021	Yes	Western blotting (n = 18) Flow cytometry (n = 10) Immunofluorescence (n = 10)	PVN Blood PVN	Cit-H3 Cit-H3, MPO Cit-H3	Elevated at 24, 28, 72, 96 h; peaks 96 h Elevated at 72 h Elevated at 72 h

*Cit-H3*  citrullinated histone H3, *ELISA*  enzyme-linked immunosorbent assay, *FACS* fluorescence-activated cell sorting, *HMGB1*  high mobility group box 1, *Ly6G*  lymphocyte antigen 6 complex locus G6D, *MPO*  myeloperoxidase, *NE*  neutrophil elastase, *N/S*  not specified, *PAD4*  Peptidyl arginine deiminase 4, *PVN*  paraventricular nucleus, *SEM*  scanning electron microscopy

*No *p* values given. Worded as: ‘relatively high’ regarding increase in percentage of NET formation in brain parenchyma and ‘more easily’ in regard to NET formation following TBI

** No *p* values given as no control group but states that as signs of NETosis demonstrated

#### NET Presence in Humans

Five studies contained a clinical component [27, 29, 33, 34, 38], in addition to reporting results from an animal model, with four studies demonstrating NET formation following TBI in humans (Table 3). One study was excluded from further analysis as the clinical component investigated the relationship between neutrophil count and Glasgow Coma Scale (GCS), with the presence of NETs not being investigated [34]. These clinical investigations were in patient blood serum specimens isolated from TBI patients [27, 29, 33, 38], with one manuscript also including brain tissue obtained from severe TBI patients who underwent craniotomy to evacuate a haematoma and/or to treat refractory raised ICP [27]. Regions of brain/contusion sample included the right parietal lobe and the right (2 ×) and left frontal lobe.

**Table 3:** NET presence in human samples

Study ID	Patient injury (n)	Severity of injury	Specimen	Patient characteristics	Comparator (n)	Comparator characteristics	NETs significantly increased vs control (p < 0.05)	Method to evidence NETs	NET locality	NET markers	Other outcomes
Summary	Of the studies that included a clinical component (n = 5), four significantly demonstrated the presence of NETs in serum samples from 156 TBI patients compared to healthy controls or patients with normal pressure hydrocephalus. Cit-H3 was the most frequently used NET marker, with a range of techniques employed to determine NET presence. The severity of injuries in TBI patients ranged from mild to severe, and the demographic details of the patients were not provided in most cases. NET presence was also significantly elevated in the brain tissue of TBI patients (n = 4)										
Cao et al., 2023	TBI (14)	Severe	Plasma from whole blood (n = 10); Human brain tissues (n = 4)	N/A for patients’ plasma; 53 years ± 7.6, 50% male	Matched healthy donor (n = 10); Human brain tissue of patients with glioma undergoing maximising resection (n = 3)	N/A for comparator plasma; 43 years ± 12.1, 66.6% male	Yes	ELISA Immunofluorescence	Plasma Brain tissue	Cit-H3, cf-DNA Cit-H3, MPO	NET quantification showed increases at lower presenting GCS; NETs quantification positively correlated with elevated ICP
Jin et al., 2023	TBI (128)	Moderate-severe	Blood	Coagulopathy – (n = 68) 35.6 ± 9.6 years, 38% male; coagulopathy + (n = 60) 33.2 ± 10.3 years, 42% male	Healthy donors (n = 34)	33 years ± 10.2, 50% male	Yes	ELISA Confocal microscopy	Plasma	Cit-H3, MPO, NE, cf-DNA MPO	N/A
Mi et al., 2023	TBI (8)	Not specified	Blood	N/A	Healthy donors (n = 8)	N/A	Yes	Western blotting DNA assay	Plasma	Cit-H3 cf-DNA	N/A
Vaibhav et al., 2020	TBI (10)	Severe	Blood	N/A	Patients with normal pressure hydrocephalus requiring routine CSF fluid diversion (n = 10)	N/A	Yes	ELISA	Plasma	MPO, DNase	Inverse correlation between serum DNase activity and ICP in patients; strong correlation between serum DNase and GCS

*cf-DNA*  cell free DNA, *CSF*  cerebrospinal fluid, *Cit-H3*  citrullinated histone H3, *DNA*  deoxyribonucleic acid, *ELISA*  enzyme-linked immunosorbent assay, *GCS*  Glasgow Coma Scale, *ICP*  intracranial pressure, *MPO*  myeloperoxidase, *N/A* not available, *NE*  neutrophil elastase, *NET*  neutrophil extracellular trap. Age data presented as median ± SD

All experiments reported statistically significant elevated NETs from serum in a total of 156 TBI patients compared to healthy controls or patients with normal pressure hydrocephalus, using a variety of NET markers and analytical techniques. Significantly elevated plasma cf-DNA [27, 29], Cit-H3 DNA [27, 29], MPO-DNA [29, 38], NE-DNA [29] and reduced deoxyribonuclease (DNase) activity [38] were observed in serum following enzyme-linked immunosorbent assay (ELISA) analysis. Consistently significantly elevated circulating cf-DNA in TBI patients’ plasma was demonstrated by a DNA assay, alongside significantly elevated Cit-H3 in the blood of TBI patients as demonstrated by western blotting [33]. Moreover, NET formation was reported to be significantly elevated in TBI patients’ serum compared to healthy controls by confocal microscopy observation [29]. Importantly, NET formation was significantly increased in TBI brain tissue over controls (brain tissues obtained from patients with glioma, with donation of perilesional tissue during resection), demonstrated by immunofluorescence with Cit-H3 and MPO markers [27]. Furthermore, elevated NETs [27, 38] inversely correlated with GCS scores on presentation, with elevated NETs indicating greater injury severity and poorer outcomes.

#### NET Modulation

After demonstrating NET formation post-TBI, 11 studies [27–30, 32–34, 36–39] went on to investigate the effect of NET modulation on functional outcomes and TBI-associated deficits in pre-clinical models. All studies investigating NET modulation used an in vivo model of TBI, with one study also using an in vitro model of co-cultured isolated serum neutrophils and microglia post-DAI [39] as an adjunct to an in vivo TBI model.

Degradation [27, 29, 30, 33, 38] or inhibition [27–30, 32–34, 36–39] were the two mechanisms used to modulate NETs. Degradation was exclusively achieved utilising DNase, while PAD4 inhibitors were primarily the mechanisms of action for inhibition, with one study alternatively using anti-HMGB1 to inhibit NETs [36]. NET degrading therapies were administered via intravenous injection. GSK484, a NET inhibitor, was also administrated in this way [34]. Cl-amidine, a PAD-4 inhibitor, was administered by intraperitoneal injection [27, 30, 32, 33, 37, 38], as was polydatin [28], while anti-HMGB1 was administered via stereotaxic in situ injection to the paraventricular nucleus (PVN) [36] and YM3-56 added to 24-h co-culture of isolated serum neutrophils and microglia after DAI [39]. A PAD4-/- knockout mouse model was additionally used [43].

All employed vehicles alongside injury as comparators. NET modulatory strategies used, including therapeutic regimen, comparators, and summary of outcomes, are detailed in Table 4.

**Table 4:** NET modulation

Study ID	NET modulation strategy	Method of administration	Dose	Dosing strategy	Comparator	Summary of outcomes
Summary	NET modulation significantly improved outcomes in all studies (n = 11) investigating its effects following TBI. DNase and Cl-amidine were the most common NET modulatory methods, and in-vivo models were used throughout. Doses were also primarily administered 1 h after injury					
Cao et al., 2023	DNase (NET degradation) Cl-amidine (NET (PAD4) inhibition)	IV injection IP injection	5 mg/kg 10 mg/kg	1 h after injury and injected daily until euthanasia 10 min after injury and once per day for 3 consecutive days	TBI with vehicle	NET modulation (Cl-amidine) significantly improved functional outcomes (*p* < 0.05) (mNSS, latency to falls) at 3- and 5-days post-injury NET modulation ameliorated neurological and pathophysiological TBI-associated deficits investigated (neuroinflammation (*p* < 0.05 at 3-days, DNase and Cl-amidine), neuronal cell death (*p* < 0.05 at 3-days, DNase), neuronal damage and necrosis (*p* < 0.001 at 3 days, Cl-amidine), ER-stress (*p* < 0.05 at 3-days, DNase, Cl-amidine), sympathetic hyperactivity)
Gu et al., 2021	Polydatin (NET inhibition)	IP injection	30 mg/kg	Immediately following injury	TBI with vehicle	NET modulation ameliorated pathophysiological TBI-associated deficits at 6 h in experimental models (cerebral oedema (histopathological changes), inflammatory markers in lungs, neuronal degeneration (lessened darker staining), TBI-induced lung injury *(p* < 0.05 for all deficits investigated, where available))
Jin et al., 2023	PAD4-/- (knockout) mice (NET inhibition) DNase (NET degradation)	C57BL/6 PAD4-/- mice obtained Tail vein IV injection	N/A 5 mg/kg	N/A 1 h before and after trauma	TBI TBI with vehicle	NET modulation significantly improved mNSS (*p* < 0.0001) for all interventions and timepoints (6, 12, 24, 48 h) PAD4-/- and DNase treated mice showed reduced 3-day mortality (44.44% to 22.22% and 55.56 to 33.33% respectively [both *p* < 0.05]) Neurological deficits investigated also improved through NET modulation (cerebral oedema (topical views of brain, PAD4-/-), BBB dysfunction (*p* < 0.05 PAD4-/-, *p* < 0.0001 DNase), coagulopathy (*p* < 0.05 for PAD4-/-, DNase)
Li et al., 2024	DNase (NET degradation) Cl-amidine (NET (PAD4) inhibition)	Tail vein IV injection IP injection	5 mg/kg 50 mg/kg	1 h after injury and then daily until euthanasia 10 min after injury and then daily until euthanasia	SBI with vehicle	NET modulation significantly improved functional outcomes (modified Garcia score; assessment of neurological function) at 3-(inhibition *p* = 0.0133, degradation *p* = 0.0091) and 7-days post-injury (inhibition *p* = 0.0109, degradation *p* = 0.0079) NET modulation ameliorates TBI-associated deficits investigated at 3 days (cerebral oedema (DNase, p = 0.0058) (Cl-amidine, p = 0.0021)); neuroinflammation (*p* < 0.05 DNase and Cl-amidine), neuronal cell death (*p* < 0.05 DNase and Cl-amidine)
Liu et al., 2022	Cl-amidine (NET (PAD4) inhibition)	IP injection	50 mg/kg	Immediately following injury and then every 3 days	TBI with vehicle	NET modulation significantly improved functional outcomes investigated (footfalls at 2 weeks *p* = 0.0482; time (s) in centre of zone at 4 weeks *p* = 0.0110 post-injury; improved survival rate at 1 week (no p value given but stated as significant)) NET modulation ameliorates TBI-associated deficits investigated (BBB dysfunction (*p* < 0.05 at 6 and 24 h)
Mi et al., 2023	DNase (NET degradation) Cl-amidine (NET (PAD4) inhibition)	IV injection IP injection	10 mg/kg 50 mg/kg	24 h after injury and injected every 12 h until sacrifice 10 min after TBI and then every day until sacrifice	TBI with vehicle	NET modulation significantly improved functional outcomes (Cl-amidine *p* < 0.05, mNSS at 7- and 14-day and latency (s) to falls on rotarod at 3-, 5-, 7-, 14-day; DNase *p* < 0.05, mNSS at 7- and 14-day and latency (s) to falls on rotarod at 7- and 14-day) NET modulation ameliorated neurological deficits investigated in TBI experimental models (neuronal cell death (*p* < 0.05 at 3 days, Cl-amidine and DNase), neuronal damage and necrosis (*p* < 0.05 at 3 days, DNase and Cl-amidine), ER-stress (*p* < 0.05 at 3-days, DNase, Cl-amidine)
Mu et al., 2024	GSK484* (GSK, NT-GSK, T-GSK) (NET (PAD4) inhibition)	Tail vein IV injection	1 mg/ml	Administered 1, 4 8 and 24-h after injury	TBI with vehicle	All NET modulatory experimental groups improved functional outcomes (mNSS and latency to falls (s); no p value given comparing vehicle controls) NET modulation ameliorates TBI-associated deficits investigated (all 3-day, all therapies)) (cerebral oedema *p* < 0.05, CBF *p* < 0.05, BBB dysfunction *p* < 0.05, neuroinflammation (*p* < 0.05), neuronal cell death (*p* < 0.05) T-GSK demonstrating the most benefit and GSK the least regarding functional outcomes and TBI-associated deficits. Acceleration of recovery observed following NET modulation
Qu et al., 2023	Anti-HMGB1 (SP60012) (NET inhibition) **	Stereotaxic injection to paraventricular nucleus	0.2μL NS	Injected before injury	DAI with vehicle	NET modulation ameliorated the neurological and pathophysiological TBI-associated deficits investigated (neuroinflammation *p* < 0.05 at 3-days; sympathetic hyperactivity)
Shi et al., 2023	Cl-amidine (NET (PAD4) inhibition)	IP injection	50 mg/kg	Administered 1 h after injury and then once per day for three consecutive days	TBI with vehicle	NET modulation significantly improved functional outcomes on 1- (mNSS *p* < 0.01 and *p* < 0.05; latency (s) to falls on rotarod test *p* < 0.05 and *p* < 0.05; corner test (assessment of postural and sensorimotor asymmetries) *p* < 0.05) and 3-day (mNSS *p* < 0.05 and *p* < 0.01; latency (s) to falls on rotarod test *p* < 0.05 and *p* < 0.05; cylinder test (behavioural tool evaluating forelimb motor function)* p* < 0.01, corner test *p* < 0.05). *** NET modulation ameliorated TBI-associated deficits investigated (cerebral oedema (*p* < 0.05 at 1-day, *p* < 0.001 at 3-day), CBF (*p* < 0.05 at 1-day, *p* < 0.01 at 3-day), BBB dysfunction (*p* < 0.05 at 1-day, *p* < 0.01 at 3-day), neuroinflammation (*p* < 0.05 at 3 days), neuronal cell death (*p* < 0.05 at 3 days), degenerating neurones (*p* < 0.01 at 3 days), neuronal damage and necrosis (*p* < 0.05 at 3 days), ER-stress (*p* < 0.001 at 3-days, Cl-amidine), sympathetic hyperactivity)
Vaibhav et al., 2020	DNase (NET degradation) Cl-amidine (NET (PAD4) inhibition)	Tail vein IV injection IP injection	5 mg/kg 10, 30, 50 mg/kg ****	Administered at 1 h after TBI Administered 10 min after injury	TBI with vehicle	NET modulation significantly improved functional outcomes (latency to falls (s) *p* < 0.05 (Cl-amidine (50 mg/kg) and DNase); time (s) spent in centre zone at 2 months *p* < 0.01 (DNase); number of slips at 2 months* p* < 0.05 (DNase); time to cross beam at 2 months* p* < 0.001 (DNase and Cl-amidine (50 mg/kg)) and* p* < 0.05 DNase); grip strength (neuromuscular function) at 2 months* p* < 0.05 (DNase); discrimination index (recognition memory) at 2 months *p* < 0.05 (DNase)) NET modulation ameliorates TBI-associated deficits investigated (cerebral oedema (*p* < 0.01 at 24 h, DNase; *p* < 0.05 at 24-h, Cl-amidine (50 mg/kg)); CBF (*p* < 0.05 1-h, *p* < 0.05 6-h, *p* < 0.05 1-day, Cl-amidine (50 mg/kg)
Zhu et al., 2021	YW3-56 (NET (PAD4) inhibition)	Added to 24-h co-culture of isolated neutrophils and microglia after DAI	5 μM	Cultured for 24 h	DAI with vehicle	NET modulation improved TBI-associated deficits investigated (neuroinflammation *p* < 0.05, sympathetic hyperactivity)

*BBB*  blood–brain barrier, *CBF*  cerebral blood flow, *DAI*  diffuse axonal injury, *DNase* deoxyribonuclease, *ER-stress*  Endoplasmic reticulum stress, *HMGB1*  high mobility group box 1, *IP* intraperitoneal, *IV* intravenous, *mNSS*  modified Neurological Severity Score, *NET*  neutrophil extracellular traps, *NT*  non targeting, *PAD4*  Peptidyl-arginine deiminase 4, *SBI*  surgical brain injury, *T*  targeting, *TBI*  traumatic brain injury

*A recently developed PAD4 inhibitory nanoparticle termed GSK484 (and variants)

**HMGB1 is an important NET component and plays a role in NET formation so anti-HMGB1 inherently NET modulatory, despite not being directly detailed for modulating NETs

***Where two p values, each representative of two separate experiments

****Dosage administered to separate animals

#### Meta-Analysis: Impact of NET Modulation on Functional Outcomes

Two outcomes were deemed suitable for inclusion in the meta-analysis: modified neurological severity score (mNSS) and latency to falls (s) on the rotarod test, using endpoints in a standardised mean difference model.

#### mNSS

**Fig. 2:**
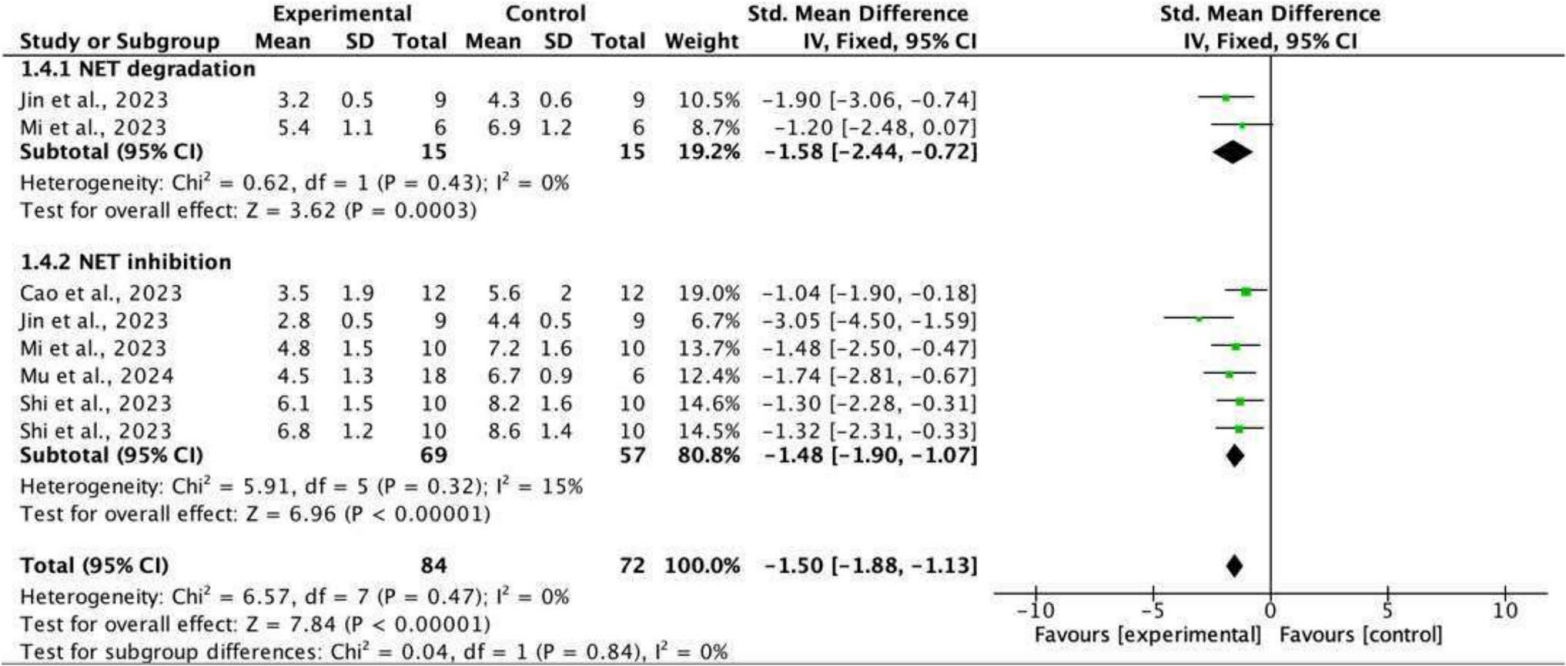
Forest plot and sub-group analysis: end mNSS

mNSS included motor, sensory (visual and tactile), reflex, and balance tests assessing neurological function. A meta-analysis was performed for the five studies [27, 29, 33, 34, 37] reporting mNSS values in experimental and control groups, with a total of eight independent experiments conducted, with varying methods of NET modulation, included in the analysis (Fig. 2). The overall effect size calculated was −1.50 (−1.88, −1.13), favouring NET modulation over control (*p* < 0.00001, I^2^ = 0%), demonstrating significantly lower mNSS, reduced neurological impairment and improved neurological function.

Given the heterogeneity regarding mechanism of action, subgroup analysis of mNSS by method of NET modulation (degradation or inhibition) was performed. Both NET degradation (*p* < 0.0003) and inhibition (*p* < 0.00001) had statistically significant effects on mNSS with no significant present difference between the two groups (*p* = 0.84) (Fig. 2).

#### Latency to Falls (Seconds)

**Fig. 3:**
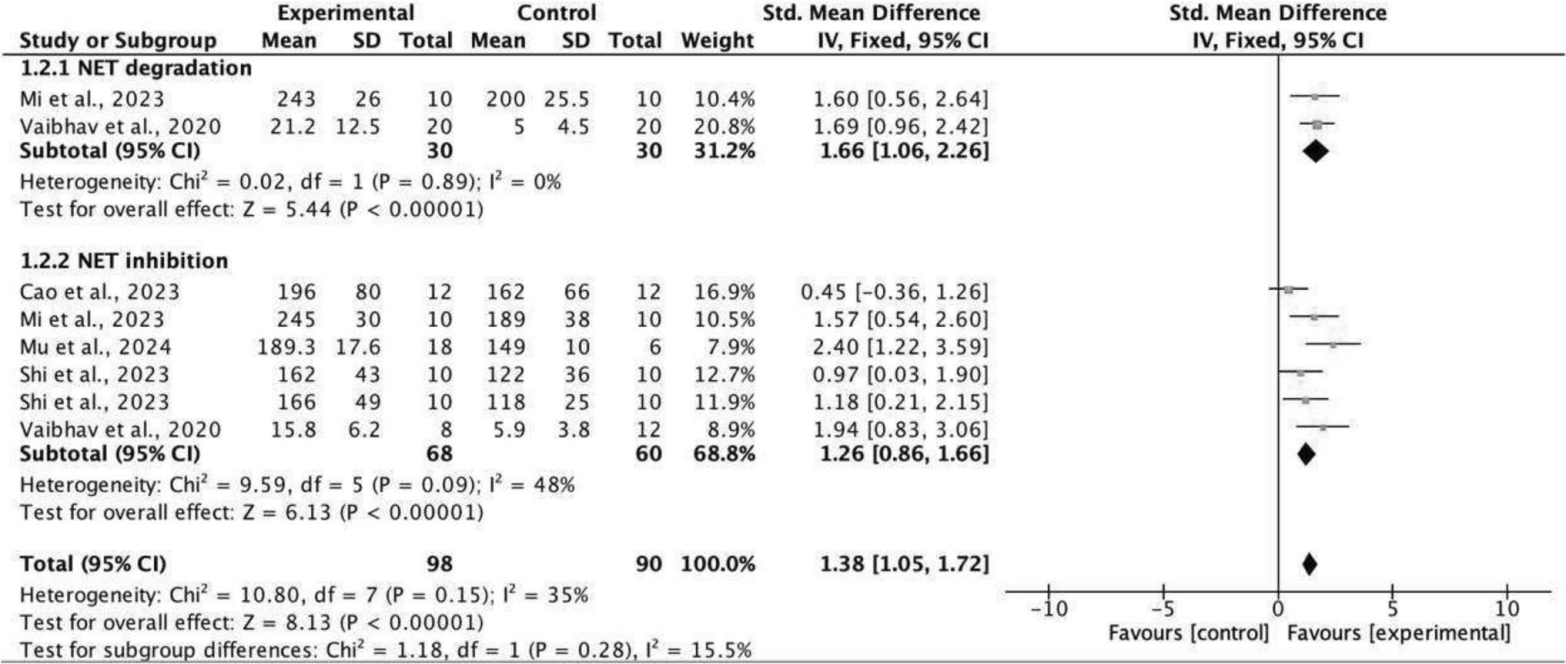
Forest plot and sub-group analysis: end latency to falls (seconds)

Fall latency on the rotarod test assesses motor coordination. Again, five studies [27, 33, 34, 37, 38], comprising eight independent experiments, reported latency to falls/time on rotarod (s) at endpoint, and were included in a meta-analysis (Fig. 3). The overall effect demonstrated favour towards the NET modulation (*p* < 0.00001, I^2^ = 35%) with an effect size of 1.38 (1.05, 1.72) demonstrating significantly improved latency to falls, motor and neurological function.

Subgroup analysis by method of NET modulation was also conducted for latency to falls. Consistently, both degradation (*p* < 0.00001) and inhibition (*p* < 0.00001) NET modulatory strategies had significant effects on improving latency to falls, with no significant difference present between the groups (*p* = 0.28) (Fig. 3).

#### Other Outcomes

Other alternative functional outcomes reported throughout the included studies, unable to be subjected to meta-analyses are detailed in the supplementary material section. The impact of NET modulation on neurological and TBI-associated pathophysiological deficits, was also investigated, with improvements in various deficits observed in the included studies upon modulation of NETs. A full breakdown and narrative synthesis of these results can be found in the supplementary materials. Additionally, the effect of NET modulation on NET formation can also be found in the supplementary results section.

Investigations into the mechanisms whereby NETs are neurodestructive and the pathways which NETs contribute towards secondary injury in TBI were also conducted. The findings of these investigations and discussion can be found in the supplementary materials.

#### Risk of Bias Assessment

**Fig. 4:**
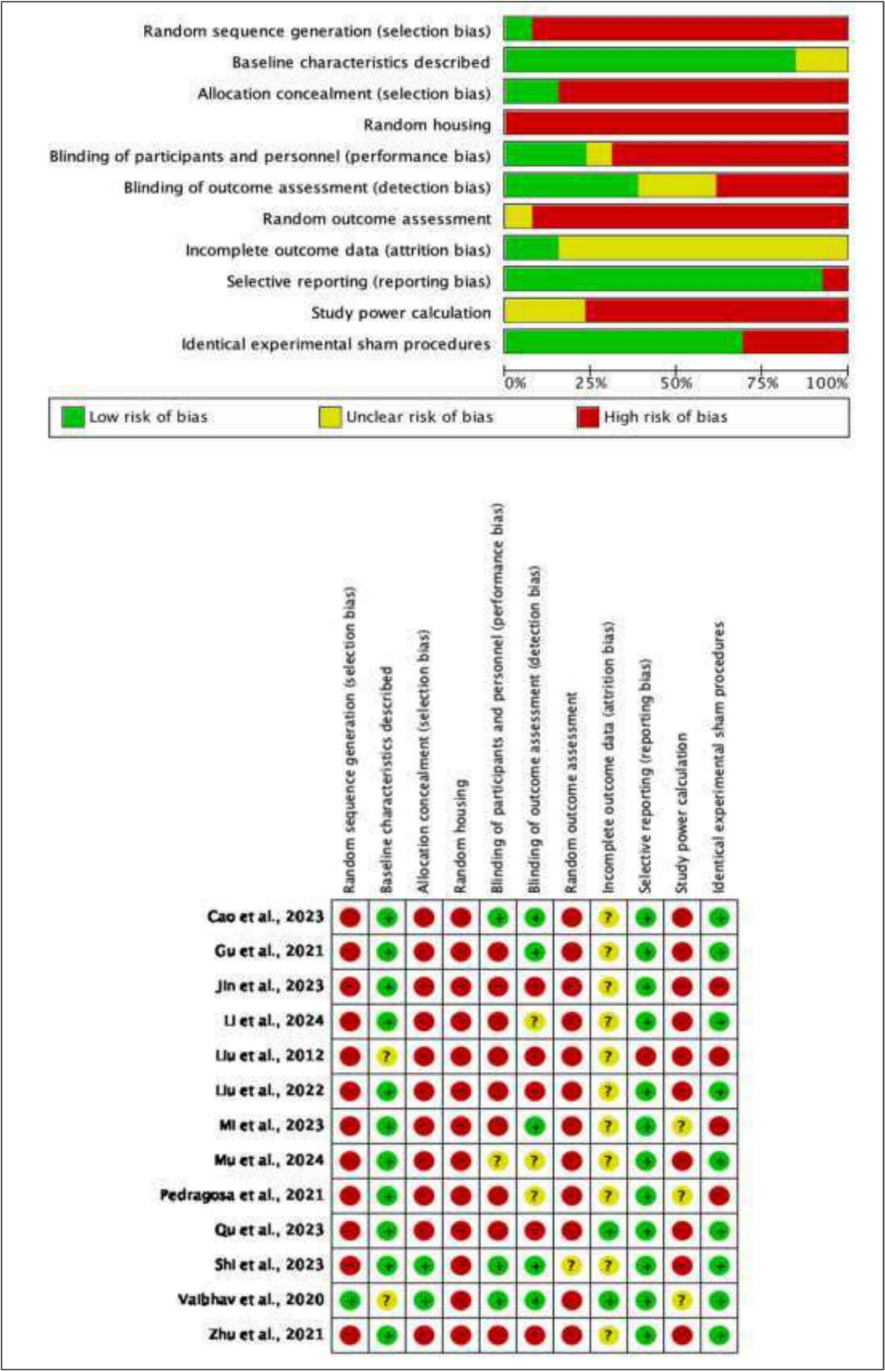
Risk of bias analysis for pre-clinical models included using the SYRCLE tool

The RoB observed across most of the included animal studies was high as detailed in Fig. 4. For the majority of studies, despite experimental subjects being described to be randomly allocated to groups, no method of sequence generation or randomisation was detailed leading a high ROB being determined for this category in studies where this occurred. The RoB analysis for included human studies is detailed in Table 5. Several studies (papers by Jin and colleagues, Mi and co-workers, plus Vaibhav and associates) were determined to contain a high ROB during preliminary screening of bias, meaning no further assessment was required, with study quality already being determined as low. An overall RoB for the included human study warranting a full assessment was determined as “fair”.

**Table 5:** Risk of bias summary for human studies*

Study ID	Cofounding factors	Measurement of exposure	Selection of participants info the study	Post-exposure interventions	Missing data	Measurement of the outcome	Selection of reporter result	Overall study quality
Cao et al., 2023	Low	Low	NI	Some concerns	Some concerns	Some concerns	High	Fair
Jin et al. 2023	High risk of bias determined during preliminary screening **							Low
Mi et al*.* 2023	High risk of bias determined during preliminary screening **							Low
Vaibhav et al*.* 2020	High risk of bias determined during preliminary screening **							Low

^*^5 studies identified contained a clinical component, as an extension of their animal model, demonstrating NET presence in humans, except for one study (Mu et al. 2024) which investigated relationship between neutrophil count and GCS and is therefore not included in this table as it was excluded from further analysis in the review, therefore being beyond the scope of the risk of bias assessment for human studies**.** **A full risk of bias assessment was not required due to a high risk being decreed during preliminary screening stages

## Discussion

This systematic review details a comprehensive summary of the available literature regarding NETs in the context of TBI. Clear evidence of NET generation after TBI is extensively demonstrated in all animal models identified and four studies provide evidence of NET formation in humans. NETs were shown to feature in the blood, brain and cerebral vasculature of animal experimental subjects following TBI, as well as the serum and brain parenchyma of TBI patients. The meta-analysis and narrative synthesis provide robust evidence that NET modulation improves functional outcomes post-TBI, validating NETs as a promising target for therapy post-TBI. Investigation into the optimal method of NET modulation was inconclusive: the available evidence supports the use of both modalities, though further direct comparative studies with appropriately powered sample sizes are required to discern any meaningful differences between approaches. Nevertheless, the findings described here support NETs as a promising therapeutic target for immune modulation in TBI to improve outcomes, warranting further investigation.

### Establishing a Translational Effect

Throughout studies included, PAD4 inhibitors, a NET inhibitor, and DNase, a NET degrader, were the most frequently used molecular approaches targeting NETosis. With DNA being the main component of NETs and owing to the presence of a chromatin backbone and histones, NETs are rendered susceptible to breakdown by DNase, an endogenous NET degrading enzyme shown to be suppressed following TBI [16, 38, 40]. PAD4 has an important regulatory role in NET formation, through mediation of chromatin decondensation via hypercitrullination of target histones, weakening the interaction of histones and DNA [13, 16, 41]. Inhibition of this mechanism therefore provides a mechanism of inhibiting NET formation. Whilst previously described in pre-clinical models, a PAD4 inhibitor has been shown to effectively reduce NET formation in human neutrophils [42]. Similarly, DNase has been shown to effectively degrade NETs released by stimulated human neutrophils in a concentration-dependent manner [43]. In the presented meta-analyses, for both approaches, NET modulation appears to improve functional outcomes after TBI in pre-clinical models. NET modulation was associated with strong recovery in motor, sensory, reflex and balance deficits in the rodent, which is sufficient to be encouraging for a clinically significant improvement in outcome in humans. However, profound effects have previously been observed in pre-clinical studies but have failed to demonstrate efficacy in human trials, with a notable example being methylprednisolone [44]. Despite this caveat, the promising data demonstrated in this meta-analysis strongly supports further investigation in translational studies.

### Establishing Optimal Therapeutic Strategy

Findings from this review suggest that future studies should also investigate optimal timing of therapy administration. The 3-day peak of NETs and the potential of NETs to last up to 14 days post-TBI provides a timely and beneficial therapeutic window of opportunity for intervention. The optimal timepoint for administration of therapies within this therapeutic window needs to be defined to maximise pre-clinical efficacy before translation into the clinical setting. Also, as early damage is difficult to mitigate due to rapid onset, NETs pose as a more effective target, as they can be targeted after longer periods. Future studies should also include therapies at a range of dosages, alongside administration timepoints and administration routes, to determine optimal dosing strategies, with only one study investigating a range of doses.

Before translation into clinical trials, sufficient pre-clinical data is required on multiple TBI models, with differing injury severities, detailing optimal therapeutic parameters to enable successful translation to humans. Dosing strategies detailed in included studies followed a schedule largely universal across the studies. DNase was generally administered at 5 mg/kg at 1-h post-trauma [27, 29, 30, 38]. Similarly, all NET inhibitory therapies were administered within 1-h post-injury. However, one study administered 10 mg/kg of DNase 24-h after injury [33]. This is particularly important as it demonstrates intervention efficacy despite delay between injury presentation and administration of therapy, meaning there is a longer window for intervention. All included studies administered therapies early after TBI or even before brain injury, which is not clinically relevant or indeed possible in most contexts of civilian TBI. Early administration of an agent in the pre-hospital setting is also difficult in practise due to difficulties in establishing a definitive diagnosis and suitability for therapies [45–47]. Hence, demonstrating efficacy of administration up to 24-h post-injury is important as it is much more reflective of an achievable clinical scenario. Future studies should aim for acute administration of agents within a more realistic timeframe; for example, in the CRASH-3 trial, tranexamic acid was administered within 3 h [48].

### Safety of NET Modulation and Practical Applications of Treatment Strategy

Immunomodulation presents a logical strategy to combat secondary injury after TBI. Despite this, few clinical trials of TBI therapies primarily targeting inflammation have been reported [49]. Of the trials with reported results, all have thus far failed to demonstrate significant clinical benefits [50, 51]. Furthermore, non-specific attenuation of inflammation using high doses of methylprednisolone has been associated with an increase in mortality post-TBI [52]. Previous studies are lacking in a targeted, anti-inflammatory strategy and individualised approach according to inflammatory biomarkers and duration of therapy [49]. Global neutrophil depletion carries the increased risk of systematic infections [53], with potentially detrimental implications in the polytrauma patient. This further emphasises the requirement for a targeted immunomodulatory therapy, selectively preserving the beneficial effect of neutrophils and an inflammatory response, while impeding deleterious effects.

Due to the ability and functionality of NETs to counter infection, there are potential concerns regarding safety of NET modulation as a therapeutic strategy in terms of immune suppression and subsequent risk of infection [54]. This is an especially important consideration in critically injured (e.g. polytrauma) patients, as often seen in TBI. However, even when unable to generate NETs, PAD4-/- (knockout) mice did not exhibit increased vulnerability to severe polymicrobial sepsis [55]. This early evidence of safety mitigates potential concerns regarding immune suppression of NETs as a treatment strategy.

DNase was one of the most common modulatory strategies used in the included studies and an aerosolised form was approved by the US Food and Drug Administration in 1993 to improve pulmonary function in cystic fibrosis (CF) patients [56], with extensive clinical safety evidence in the context of other diseases including CF [57] and COVID-19 pneumonia [58]. PAD4 inhibition was the other most frequent molecular modulatory strategy used to modulate NETs within the studies identified in this review. However, currently no PAD4 inhibitors have been approved for human use.

### BBB Penetration, Delivery to Damaged Brain Area and Future Applications

The significant damage to the BBB and loss of permeability during TBI can be exploited to deliver therapeutics that under normal circumstances are unable to cross the BBB [59]. There is evidence demonstrating the precise targeting of the nanoparticle GSK484 (targeted-GSK) to damaged brain tissue in an included study [34]. This is an important finding as therapeutics are rarely assessed in terms of brain penetration and distribution, when administered systemically [45, 60]. More studies on brain penetration and distribution are therefore required to understand the pharmacodynamics of different NET modulatory strategies following systemic administration to determine whether they successfully cross the BBB and reach damage brain tissues to exert an effect on lesion. Alternatively, rather than harnessing BBB damage to deliver therapeutic agents, where inherent heterogeneity issues between patients preside regarding scale of damage and time to endogenous BBB repair, a recent TBI mouse model was successful in delivering a therapeutic agent using a nanoparticle across the BBB, breaching independently of damage providing a promising approach for delivery of therapeutics to the brain in TBI patients [59, 61]. Delivery of NET modulatory agents in this way may provide an innovative strategy enabling a greater improvement of TBI outcomes, overcoming some potential limitations of drug delivery to the brain.

### NET Markers

Several markers can be utilised to detect NET formation including Cit-H3, MPO, NE, and cf-DNA, amongst others. However, cf-DNA may potentially be derived from other non-NET sources, including necrosis and release from macrophages [14] and therefore is considered a non-specific NET marker [62]. Conversely, with citrullination of histones by PAD4 being a crucial element in NET formation, Cit-H3 is considered to be a more specific, objective and highly quantitative NET marker [63]. However, as Cit-H3 is only produced during PAD4-dependent NETosis a limitation presides, meaning this marker is not present in and cannot be detected in all form of NETosis [62, 63]. MPO and NE DNA complexes are highly specific, objective and quantifiable markers [62, 63], as these proteins appear to be present across the differing types of NETosis and are vital components in NET formation [64, 65]. To allow effective delivery of NET modulatory therapies, rapid prognostication confirming NET presence in TBI patients using specific, objective and quantitative markers is required. This will facilitate the personalisation of approaches and determine the dose and duration of therapies required based on NET-specific biomarkers.

### Controls

In humans, plasma NETs may potentially be manifestations of trauma elsewhere, derived from sources outside the central nervous system. TBI patients commonly present with trauma in other locations outside the BBB, which will leak inflammatory markers more readily into the blood, and therefore serum NET markers are highly unlikely to be specific to TBI. The use of appropriate controls (e.g. non-TBI trauma patients) is vital to investigate these likely limitations, however all the studies discussed investigating serum NETs utilised healthy controls, which does not elucidate whether such markers are TBI specific. As NETs appear to be a feature in other general trauma [66], use of serum controls will not allow for the specific attribution of markers to TBI when compared to healthy volunteers. There is therefore a requirement for serum controls to be healthy controls compared with trauma patients injured exclusively to the brain. Alternatively, obtainance and comparison of brain tissue specimens from TBI and non-TBI patients will also allow the attribution of NETs as a specific feature of TBI. Cao and colleagues used these appropriate controls in the form of glioma patients undergoing maximising resection as their non-traumatic brain tissue specimens, making this demonstration of NETs in TBI patients brain tissues a crucial finding [27].

### Risk of Bias

Several studies were evaluated to be at a high RoB, which hinders the ability to draw reliable conclusions from the review and meta-analysis findings. Factors were not reported as per SYRCLE guidelines in pre-clinical models, and most studies commonly overlooked study power calculation, random sequence generation and blinding. These lapses in methodical rigor may lead to the effect size of NET modulation in the meta-analyses being overestimated, ultimately affecting the findings'practical significance. Underpowered studies due to small sample sizes may overestimate the magnitude of the true effect because of the greater random variation in the results. Randomisation issues and lack of blinding can contribute to this by failing to control confounding variables properly and allowing biases to occur, which inflates the effect size. While some aspects of the RoB assessment are not required to be implemented or reported in animal models [25], future studies should improve study design in areas identified at a high RoB to mitigate these issues, with closer adherence to SYRCLE guidelines, with specific attention to study power, randomisation, and blinding. There is also an indeterminate risk for publication bias in the literature, which may have influenced the perceived effects found in this meta-analysis. Improved practices in reporting of negative results for any future NETS studies in TBI is encouraged. Clinical studies could be improved by reporting all outcome data and full transparency of results, enabling valid conclusions to be drawn.

### Limitations

A fundamental limitation of this study is the lack of clinical trials, with the presented results primarily based on pre-clinical experimentation. Heterogeneity in data presentation, outcome measures, and method used to evidence changes prevented a meta-analysis from being conducted for outcomes other than mNSS and latency to falls. As a result, the scope of the meta-analysis was limited to these two common outcomes. Additionally, there are intrinsic biases towards a specific subset of publications due to the availability of outcome measures (mNSS and latency to falls) within the literature and biases introduced due to omitting other outcomes from a meta-analysis.

The predominance of severe models within the review, also necessitates the requirement for additional studies involving a wider range of severity types and comparisons between severities. Further exploration into mild-moderate models will facilitate more robust conclusions to be drawn regarding potential therapy efficacy in mild-moderate injury and suitability at different severities.

Another limitation of the studies which used DNase as a NET modulatory strategy is that DNase degrades multiple forms of DNA, not just from NETs [30]. Therefore, when used as a NET modulating strategy, DNase may be degrading off target DNA sources, potentially mediating observed improvements, incorrectly attributing it to NET degradation [38]. Although in many of the studies decreases in NETs were verified post-modulation, the improvements may not be solely due to NET degradation, blurring the perceived benefit of this therapeutic strategy. Also, as NETs have the potential to form via PAD4 independent mechanisms, this potentially renders PAD4 inhibitors ineffective in these situations. Further investigations into the type of NETosis occurring following TBI is required to facilitate the administration of appropriate therapy in line with the type of NETosis present and overcome shortcoming of NET modulatory strategies.

A limitation specific to animal models is that the animals were predominantly of a similar age, with ages mostly ranging from 8 to 10 weeks, and of similar sex, with all but one study using males. In both humans and animal models, sex differences exist in outcomes after TBI [67], and outcomes of TBI also vary with age [68]. NETs have previously been associated with hormone-dependent inflammatory processes, including oestrogen-dependent intracranial aneurysm rupture [69]. Therefore, understanding the impact of hormonal factors and sex is integral to further translation.

### Future Directions

Initial human trials utilising well-defined NET-specific biomarkers should be conducted to assess NET activity following TBI. The use of appropriate controls in future studies is essential to enable the characterisation of serum NET markers as either a specific feature in TBI patients or a feature of the general trauma response. Further study with comparison between patients with isolated TBI and those with polytrauma but without TBI may yield reliable TBI-specific biomarkers of NET activity. This could be supported by further studies examining brain tissue from patients with and without TBI, which, despite their inherent practical challenges, would be of high value in determining both the role of NETs as a specific feature of TBI in humans as well as potential novel serum biomarkers. Identification of reliable markers of NET activity would also be of significant value toward translational studies of experimental drugs aiming to modulate NET activity: such biomarkers would provide a means to validate the biological effects of NET modulators in the clinical setting as an early indicator of potential for efficacy in early-stage clinical trials.

Another area for future investigation is developing NET-targeting nanotherapeutics that can penetrate the BBB. Whilst loss of BBB integrity is a common feature of TBI, BBB-penetrant therapeutics would be beneficial to ensure reproducible effects between patients, enabling effective access to target tissue and maximising potential for NET modulation to demonstrate benefit in improving outcomes. Early attenuation of neuroinflammation should be regarded as a crucial target not only for TBI recovery but also for preventing secondary effects of traumatic neuroinflammation such as the increased risk of stroke [70]. Further investigation into the potential role of NETs in elevating post-TBI stroke risk is therefore warranted.

Numerous challenges accompany the transition from animal models to clinical applications. Animal models cannot accurately reflect complex pathophysiological changes or replicate the conditions that occur in humans following brain injury, such as hypotension, hypoxemia or systemic inflammation [71]. Nonetheless, they are necessary for elucidating the mechanisms of pathophysiological change and serve as important precursors to clinical trials, helping to guide trial parameters and ensuring safety and efficacy [45]. Further research should prioritise larger, sufficiently powered, rigorously conducted pre-clinical studies investigating NET modulation that accurately reflect clinical scenarios and mimic the complex pathogenesis of TBI in humans—including representation of diverse populations by age and sex, varying TBI severities, and a comparison of NET modulatory approaches. These studies should involve assessments of clinically relevant and salient long-term physiological, functional and cognitive outcomes to validate treatment efficacy over this period. Through a combination of accurate biomarkers, improved delivery and optimised treatment protocols, further pre-clinical work processes have significant potential to inform the design of high-quality early-phase clinical studies.

## Conclusions

This systematic review demonstrated evidence that [1] NETs appear to be a feature of inflammatory responses after TBI in both patients and animal models; [2] modulation of NETs improves functional outcomes in pre-clinical experiments; and [3] there is insufficient data to determine the most effective approach for NET modulation to improve recovery in TBI. However, the high risk of bias in the included studies limits their value. Whilst NETs may present an encouraging therapeutic target based on the evidence collated here, there is a requirement for more high-quality pre-clinical data elucidating the optimal therapeutic strategies and parameters to inform future translational studies. Due to the lack of clinical studies, the clinical significance of NET modulation as a therapeutic strategy is currently unclear and requires elucidation, with further translational research needed. Overall however, NETs present a highly promising therapeutic target in TBI by their potential to be a discrete target for modulation to limit harmful sequelae of immune and inflammatory responses to injury without compromise of overall immune function, which is integral to the acute recovery after trauma.

## Supplementary Information

Below is the link to the electronic supplementary material.

Supplementary file1 (DOCX 321 KB)

## Data Availability

All data generated or analysed during this study are included in this manuscript or supplementary materials.

## Abbreviations

BBB: Blood–brain barrier
CCI: Controlled cortical impacts
cf-DNA: Cell-free DNA
CI: Confidence interval
Cit-H3: Citrullinated histone H3
CF: Cystic fibrosis
DAI: Diffuse axonal injury
DNase: Deoxyribonuclease
ELISA: Enzyme-linked immunosorbent assay
GCS: Glasgow Coma Scale
HMGB1: High-mobility group box 1
ICP: Intracranial pressure
mNSS: Modified neurological severity score
MPO: Myeloperoxidase
NE: Neutrophil elastase
NET: Neutrophil extracellular trap
PAD4: Peptidyl-arginine deaminase 4
PVN: Paraventricular nucleus
RoB: Risk of bias
PRISMA: Preferred Reporting Items for Systematic Reviews and Meta-analyses
SD: Standard deviation
SEM: Standard error of the mean
TBI: Traumatic brain injury
TLR4: Toll-like receptor-4
