# Supplementary material for: Investigating the Role of Neutrophil Extracellular Traps as a Therapeutic Target in Traumatic Brain Injury: a Systematic Review and Meta-analysis
Source: Mol Neurobiol. 2025 May 23;62(11):14923–46. doi: 10.1007/s12035-025-05053-7 (PMC12511129; doi:10.1007/s12035-025-05053-7)
Supplement: Supplementary file 1 — Supplementary file1 (DOCX 321 KB) [file 12035_2025_5053_MOESM1_ESM.docx]

**Supplementary Material**

**Supplementary results**

This section contains aspects excluded from the main body, but that remain relevant and important to the subject matter, supporting the conclusions of the paper and adding additional understanding to the topic. A wide variety of aspects and themes from included papers have been collated and summarised in this section.

Findings revealed that neutrophil extracellular trap (NET) modulation led to improvements in a range of alternative acute and chronic functional and neurobehavioral outcomes as well as a range of neurological and traumatic brain injury (TBI)-associated pathophysiological deficits, including cerebral oedema, cerebral perfusion, blood-brain barrier (BBB) disruption, neuroinflammation, cell death and more. Supplementary Table 1 provides a summary and overview of the outcomes described in the supplementary materials, with a supplementary narrative below providing the full detail of findings alongside in further detail in Table 4 (main text).

***Alternative functional outcomes associated with NET modulation.***

Various alternative functional outcomes other than modified neurological severity score (mNSS) and latency to falls on the rotarod test concerning impact of NET modulation were reported throughout the included studies. However, lack of sufficient data (quantity and reporting methods), insufficient methodological detail (e.g., training schedules), heterogeneity in timing of testing (2 weeks to 2 months), outcome analysis used, and differing units of measurement prevented conduction of a meta-analysis for these other functional outcomes. As such, alternative functional outcomes reported are described in the outcome section of Table 4 (main text) and the narrative below.

Both acute and chronic functional and neurobehavioral outcomes are significantly improved following NET modulation vs brain injured vehicle controls. Statistically significant reductions in anxiety upon NET modulation, with time in centre of zone as proxy, at 1 (1) and 2 months (2) were observed. Following NET modulation statistically significant improvements in motor function and coordination were also observed with decreased number of slips at 2 months (2) and reduced footfall percentage at 2 weeks (1). There was a statistically significant improvement in survival rate at 3 days (3) and 1 week (1). Furthermore, statistically significant improvements in neuromuscular function (grip strength), motor coordination (time to cross beam), and recognition memory (discrimination index) all at 2 months (2) were shown, as well as time to cross beam acutely at Day 3. Statistically significant improvements were also observed following NET modulation in the corner test, assessing postural and sensorimotor asymmetries, at 1- and 3-days (4) and similarly, improvements in the cylinder test, a behavioural assessment tool evaluating forelimb motor function, were shown at Day 3 but not at Day 1 (4).

Li and colleagues (5) reported on the modified Garcia score, a score similar to mNSS assessing neurological function, to measure neurological deficits and as such could not be included in the meta-analysis. However, significant improvements in the modified Garcia score following NET modulation by both deoxyribonuclease (DNase) and Cl-amidine at both Day 3 and 7 was observed, providing further evidence of NET modulation improving neurological function.

***Impact of NET modulation on neurological and TBI-associated pathophysiological deficits.***

Similarly to functional outcomes, changes in various neurological and TBI-associated pathophysiological deficits were reported in included studies following NET modulation. Large heterogeneity in data presentation and outcome measures made conduction of meta-analyses unsuitable for any outcomes regarding impact of NET modulation on TBI-associated pathophysiological deficits. However, reported impact of NET modulation on pathophysiological deficits investigated in the included studies are described in a narrative synthesis below and in the outcome section of Table 4 (main text).

*Cerebral oedema*

Significant improvements in cerebral oedema were observed following NET modulation at 24-hours (2,4) and 3-days (4,6) vs vehicle controls detected through magnetic resonance imaging quantification and analysis. Further experimentation also showed amelioration in cerebral oedema following NET modulation vs vehicle controls via reduced brain water content at 3 days (6), histopathological changes in the injured-side cortex brain of experimental subjects at 6 hours (7), and topical views of experimental subjects’ brain (3).

*Cerebral blood flow and perfusion*

Significant improvements in cerebral blood flow (CBF) were observed by laser speckled contrast imaging at 1-hour (2), 6-hours (2), 1-day (2,4), and 3-days(4) after NET modulation vs. vehicle control. Also, following staining and imaging of cerebral tissue, quantitative analysis demonstrated a significant reduction in infarction volume at 3-days following NET modulation vs vehicle controls (6).

*BBB disruption and dysfunction*

A significant amelioration in BBB disruption and dysfunction was observed upon NET modulation vs vehicle controls, demonstrated by significantly reduced extravasation of Evans blue dye in brain parenchyma (3), notably at 6-hours (1), 1-day (1,4) and 3-days (4,6), exhibiting reduced BBB permeability and damage. The endothelial junction protein ZO-1+ was also shown by immunofluorescence to be significantly improved following down regulation after TBI upon NET modulation in the contused cortex at Day 3 (51). Moreover, Western blotting analysis demonstrated a significant improvement in down regulated tight-junction proteins, ZO-1, VE-cadherin and Occludin at Day 3, alongside reduced perivascular IgG deposits at Day 1 exhibited by immunostaining, upon NET modulation vs vehicle controls (4). Also, fluorescence-activated cell sorting analysis exhibited significant decreases in quantities of infiltrated neutrophils and CD11b+ pericytes, characterised by increased permeability, in injured brain tissues of experimental subjects upon NET modulation vs controls (1).

*Neuroinflammation*

Neuroinflammation was shown to be ameliorated following NET modulation vs controls with significant reductions in upregulated proinflammatory cytokines IL-18 (8), IL-6 (5,6), TNFα (4–6,9), IL-1β (4,8–10), iNOs (45,46) and CD86 (4) exhibited in brain tissues. A significant reduction in TNFα, IL-6, MCP-1, and IL-1β in the lungs were also observed following NET modulation vs vehicle controls (7). Significant upregulation of anti-inflammatory M2 phenotypic markers Arginase-1 (45,46) and CD206 (4) were also observed in the brain parenchyma of experimental subjects vs vehicle controls at Day 3. Significant attenuation of amplified microglia (Iba-1 expression) (4–6), astrocytes (GFAP expression) (4–6), neutrophils (Ly6G (4,5) and MPO (4) expression) and microphages (F4/80) (4) in the injured encephalon of subjects vs vehicle controls at Day 3 was also reported following NET modulation. A significant reduction in CD16+Iba+ and iNos+Iba+ M1 microglia (9), alongside significantly increased Arginase-1+Iba+ M2 microglia/macrophages (4,9) in brain parenchyma of experimental group vs vehicle control was shown, providing further evidence of NET modulation improving the inflammatory profile towards a M2 phenotype.

*Neuronal cell death*

Neuronal cell death is another neurological deficit in which NET modulation was shown to improve, demonstrated via a significant reduction in upregulated apoptotic (TUNEL+) neurones in the peri-contused brain following NET modulation vs vehicle controls (5,6,8,11), specifically regarding hippocampal CA1 and ipsilateral CTX regions (4). Furthermore, caspase-12, an endoplasmic reticulum (ER)-stress mediated apoptotic biomarker, was also significantly reduced in neurones following NET modulation vs control (11). Moreover, a significant increase in neuronal pyroptotic related proteins, Caspase-1 and N-GSDMD, (8) was observed and apoptotic signalling molecules, ASC (8), C-Caspase-3 (4,11), CHOP (11), Bax (4,11), and PARP-1 (11), were significantly down regulated following NET modulation vs vehicle controls observed at 3-days.

*Degenerating neurones*

Significant reductions in the number of degenerating neurones in the contused cortex was observed following NET modulation vs controls (4). Reduced neuronal degeneration was also observed via reduced extent of darker staining following haematoxylin and eosin staining following NET modulation vs controls. (7).

*Neuronal damage and necrosis*

The amount of neuronal damage and necrosis observed in the peri-injured cortex was significantly reduced, as demonstrated by Nissl staining (8,11), as well as neuronal tissue loss in coronal sections (4), following NET modulation vs vehicle controls. Moreover, immunofluorescence demonstrated a significant reduction in neuronal loss post TBI following NET modulation vs appropriate controls, specifically in hippocampal CA1 and ipsilateral CTX regions (4).

*Endoplasmic reticulum stress*

Endoplasmic reticulum-stress (ER-stress), a broad regulator of neuroinflammation and neuronal death, was demonstrated to improve through a significant reduction in ER-stress associated proteins p-IREα/IREα (4,8,11) and GRP79 (11), alongside a reduction in p-IREα/IREα+ neurones (4,11) and IREα+ microglia (4), upon NET modulation vs vehicle controls.

*Sympathetic hyperactivity*

Another TBI related deficit which NET modulation can ameliorate is sympathetic hyperactivity. Important pro-inflammatory mediators TNF-α and IL-1β released after TBI have been shown to participate in sympathetic hyperactivity in vitro (12,13), with IL-1β participating via regulating levels of neurotransmitters and neuronal activity (13,14) Moreover, elevated IL-1β in the paraventricular nucleus (PVN) have been evidenced to contribute to the occurrence of sympathetic hyperactivity in myocardial infarction (13), hypertension (15), and heart failure (16). Considering this, these inflammatory cells can be supplemented for markers of increased sympathetic hyperactivity, and thus NET modulatory strategies reducing these markers subsequently demonstrate apparent reduction in sympathetic hyperactivity, with statistically significant reductions of markers in brain tissues following NET modulation previously described (4,8–10). Furthermore, significantly reduced levels of serum catecholamine, mean arteriole pressure, and heart-rate variability (indicators in sympathetic excitation) in brain injured experimental subjects 3 days after injury post NET modulation vs vehicle controls was demonstrated (9). Taken together these findings indicate reduced sympathetic hyperactivity in TBI-experimental subjects vs controls following NET modulation.

*TBI-induced lung injury*

Significant improvements in TBI-induced lung injury following NET modulation were demonstrated by improved lung vascular permeability recovery, ameliorated pulmonary oedema, reduced thinner alveoli walls, alongside less erythrocytes and secretions in respiratory bronchiolar vs vehicle controls (7)

*Coagulopathy*

Significantly reduced fibrin deposition, used as a marker of coagulopathy, following elevation in lung and kidney microvasculature, alongside reduced elevated plasma D-dimer levels and improvements in reduced plasma fibrinogen levels were reported upon NET modulation vs vehicle controls (3).

***NET reduction following NET modulation***

Various changes in markers of NET formation were also observed upon NET modulation post TBI in the lungs, blood and brain of experimental subjects, with similar methods and markers to quantify this as used in experiments demonstrating NET formation.

A statistically significant reduction in NET formation in the lungs vs vehicle controls was observed upon NET modulation utilising Western blotting, with myeloperoxidase (MPO), neutrophil elastase (NE) and citrullinated histone H3 (Cit-H3) as markers (7). Statistically significant reductions in plasma NET formation were also observed following NET modulation vs vehicle controls using enzyme-linked immunosorbent assay (ELISA) with significantly reduced Cit-H3 observed 3-hours (3), 6-hours (3) and 1-day post injury (5). Moreover, plasma MPO-DNA was also significantly reduced upon NET modulation vs vehicle controls (5). Flow cytometry analysis demonstrated a significant reduction in cellular expression of MPO and NE on Lg6G+TLR4+ neutrophils (2).

Importantly, significant decreases in markers of NET expression in the brain following NET modulation were reported using immunoblotting, immunofluorescence and ELISA. Immunoblotting revealed significant reductions in Cit-H3 expression in the peri-contused cortex of subjects 3 days post brain injury following NET modulation vs vehicle controls (4,5,11). However, one Western blotting analysis failed to show statistically significant reductions in MPO expression upon NET modulation following brain injury (11). Immunofluorescence revealed significant reductions in Cit-H3 (4–6,11) and MPO (4–6) in the brain following NET modulation on Day 3 following brain injury vs vehicle controls. Flow cytometry analysis again revealed significant reductions in MPO and Cit-H3 NET markers in the brain upon NET modulation following brain injury vs vehicle controls (6). Consistently decreased Cit-H3 and MPO expression on Ly6G+ neutrophils in the brain tissue 1-day post injury of experimental subjects were observed following NET modulation vs vehicle controls (2).

Together these results demonstrate the success of NET modulatory strategies and validate efficacy of therapies, with NET modulation reducing NETs in both the blood and brain following TBI.

***Mechanistic outcomes***

Many of the included studies in this systematic review incorporated elements whereby the neurodestructive effect of NETs were investigated, including the mechanisms and pathways by which they contribute towards TBI secondary injury. Evidence of NET formation in areas of tissue injury suggests a link with brain tissue damage (17), and NETs have been shown to exacerbate and be linked to many TBI-associated pathophysiological deficits. The following narrative provides detail regarding mechanistic investigations of studies included within the review into NETs’ role in the pathogenesis of secondary pathophysiological deficits.

*Cerebral oedema*

Significant positive correlations between NETs and intracranial pressure (ICP) were observed in humans indicating potential contribution of NETs to cerebral oedema (2,8). Moreover, NETs were identified as mechanically distinct mediators of cerebral oedema formation and hypoperfusion following experimental TBI, with subjects who exhibit diminished capacity to generate NETs displaying improvements in cerebral perfusion and reduced oedema vs wild-type subjects (2). Within the same study, NET localisation was observed in parallel with a significant reduction in CBF, persistent hypoperfusion, tissue hypoxia and reduced brain tissue oxygenation. Furthermore, the reduction in cerebral oedema upon NET modulation as described previously also suggests a contribution of NETs towards this secondary deficit, however the molecular mechanisms by which NETs may contribute remain undefined. Further studies are required which investigate the molecular underpinning of NETs’ contribution to cerebral oedema.

*Pericyte appearance, BBB dysfunction and neuroinflammation*

Brain pericytes are indispensable parts of the BBB and, alongside lymphocyte infiltration, are important contributors towards the pathophysiological processes of TBI (1). Histones from NETs were shown to induce CD11b+ pericyte appearance, which exhibit a pro-inflammatory profile and are characterised by increased permeability, via interactions with dectin-1 in a protein kinase (PKC)-c-Jun dependent manner, resulting in BBB disruption and neutrophil infiltration which are important pathophysiological mechanism of TBI (1). Notably however, the ability of histones alone to promote CD11b+ expression was lower in comparison to that of a NET medium, demonstrating that there are other potential NET components which contribute to CD11b+ expression (1), warranting further exploration into other potential possible components.

*Microglial polarisation and sympathetic hyperactivity*

Other NET components have been shown to mediate other TBI-associated deficits, through promoting a pro-inflammatory environment via polarising microglia towards a M1 phenotype and facilitating the release of pro-inflammatory factors. The NET component human cathelicidin LL-37 (LL37) was shown to interact with purinergic receptor P2x7 (P2x7) in microglia to promote the secretion of IL-1β via the Hippo/mammalian sterile-twenty-like kinase (MST) pathway (10). This suggests the potential for NETs to be distinct mediators of sympathetic hyperactivity, with a correlation observed between NET concentration in the PVN and blood catecholamine, a sympathetic hyperactivity marker, through inducing the release of this cytokine (10). High-mobility group box 1 (HMGB1) from NETs was also shown to contribute towards the phenotypic conversion of microglia towards an M1 phenotype via the mitogen-activated protein kinase (MAPK) (phosphorylated Jun N-terminal kinase (p-JNK)/JNK)/activator protein 1 (AP1) (phosphorylated – Jun proto-oncogene (p-c-JUN)/c-JUN and phosphorylated–proto-oncogene c-FOS (p-c-FOS)/c-FOS) pathway, causing subsequent release of IL-1β and TNFα, resulting in observed increases in sympathetic hyperactivity (9). Within this study, levels of microglial inflammatory factors correlated with NET formation and HMGB1 levels following brain injury as well as alongside sympathetic hyperactivity (9). Moreover, HMGB1 from NETs was shown to promote the phenotypic switch of microglia to M1 via exhibition of increased pro-inflammatory markers and reduced anti-inflammatory markers in brain injured subjects and in isolated microglia upon stimulation with HMGB1 vs appropriate controls. Following probing of the signaling pathway responsible for inflammatory regulation and microglial polarisation, the MAPK pathway, p-JNK/JNK molecules increased following HMGB1 stimulation of microglial culture demonstrating contribution towards microglial polarization. AP1 genes were also demonstrated to contribute towards microglia activation with increased c-c-JUN/c-JUN and p-c-FOS/c-FOS expression following addition of HMGB1 to culture of microglia. JNK and AP1 inhibitors were subsequently shown to reduce pro-inflammatory factors and HMGB1-induced secretion of IL-1β and TNFα. Importantly, anti-HMGB1 and AP-1 inhibitors were effective in reducing sympathetic excitability after DAI (9). Together this suggests a mechanism of action whereby NETs exert effects and potential pathways by which NET modulation can effectively curtail deficits.

*The STING protein*

DNA released by NETs is one of the main sources of cell-free DNA (cf-DNA) after TBI and is a major component characteristic of NETs. Release of cf-DNA activates a series of cascades subsequently activating the transmembrane ER adaptor protein, stimulating interferon genes (STING), which plays an important role in neuroinflammation and neuronal cell death (4,5,8). STING has been shown to significantly increase in neurons and microglia post TBI, significantly correlate with NET formation, and be significantly reduced upon NET inhibition, indicating STING participation in NET-mediated downstream pathway alterations and regulation of deficits after TBI (4,5,8). Also, inhibition of an upstream STING activator protein (cyclic guanosine monophosphate-adenosine monophosphate synthase (cGAS)) alleviated brain injury induced deficits, demonstrating how the cGAS-STING pathway plays a crucial role in the pathophysiology of secondary brain injury. Moreover, pre-treatment with cyclic guanosine monophosphate–adenosine monophosphate (cGAMP), an important upstream STING signalling activator protein, reversed the effects of NET inhibition (4,5), demonstrating NET participation in secondary brain injury through activation of STING-related signalling pathways.

*Microglial mediated neuroinflammation and neuronal cell death via the STING pathway*

NETs were shown to exert neurodestructive capabilities through regulation of STING-dependent inositol-requiring transmembrane kinase/endoribonuclease 1 alpha (IRE1α)/apoptotic signal-regulating kinase 1 (ASK1)/JNK pathways (4). IRE1α is an ER sensory protein, associated with ER-stress, and a broad regulator of neuroinflammation and neuronal cell death. STING-mediated ER-stress was shown to be a mechanism by which NETs induce and regulate these neurodestructive deficits following TBI. Reduced levels of elevated pathway signalling proteins, p-IRE1α, p-JNK, and p-ASK1, alongside reduced IRE1α positive neurones and microglia, as well as inhibited IRE1α activation-associated neuronal cell death and neuroinflammation were observed following NET modulation in a STING-dependent and ER-stress associated manner following TBI (4). Notably, JNK was demonstrated to again be an important signalling protein involved in NET mediated microglial activation, with a role in neuronal cell death also elucidated here. Importantly, overexpression of PAD4 was shown to induced increased NET formation and augment the effect of NETs on deficits (i.e. further upregulate M1 microglia, reduce M2 microglia, and increase apoptotic neurones) inducing worse outcomes in TBI experimental subjects, but STING antagonist, C-176, significantly rescued these effects, with reduced levels of upregulated signalling proteins (p-IRE1α, p-JNK, and p-ASK1) observed. The ER-stress molecule IREα was verified to have an important function in neuroinflammation and neuronal death, alongside the other signalling molecules, as when inhibited neurological function and deficits improved. Taken together this confirmed a definitive role for NETs and their formation, orchestrated by PAD4, exerting neurodestructive effects after TBI through STING-mediated IRE1α activation in microglia and neurones resulting in subsequent neuronal cell death and microglial induced neuroinflammation via the STING-dependent IRE1α/ASK1/ JNK pathway (4). Moreover, through NET modulation neural damage is lessened by regulation of the same STING mediated ER-stress pathway following TBI (4).

*Neuronal pyroptosis and neuroinflammation via the STING pathway*

Nucleotide-binding oligomerization domain (NOD)-like receptor pyrin domain containing 1 (NLRP1) is an inflammasome primarily expressed in neurones, closely related to and mediator of neuronal pyroptosis, an inflammatory form of cell death triggered by proinflammatory signals and releasing pro-inflammatory molecules (8). NETs were shown to exert neurodestructive effects by inducing inflammasome-dependent neuronal pyroptosis through a STING-dependent mechanism similar to that previously described (8). NET formation was shown to be closely related to NLRP1-mediated neuronal pyroptosis post TBI and NET modulation was demonstrated to alleviate neuronal pyroptosis, both though STING-dependent mechanisms (8). NET modulation decreased upregulated pSTING expression in neurones post TBI, the number of NLRP1 positive neurones, p- IRE1α expression, and neuronal pyroptosis associated protein products, with cGAMP pre-treatment reversing these effects. This demonstrates how NETs can cause, and NET modulation can alleviate neuronal pyroptosis through the ER-stress dependent STING-IRE1α-NLRP1 pathway and that these pathway proteins are important mediators in neuronal pyroptosis. Moreover, overexpression of PAD4 and subsequent NET formation augmented the effects of NETs on deficits, including increased ER-stress and neuronal pyroptosis, alongside worse neurological function, with increases in the pathway proteins observed, demonstrating a key role for PAD4 (and NETs) in NLRP1-induced neuronal pyroptosis, exerting effects through STING-mediated ER-stress and the STING- IRE1α-NLRP1 pathway (8). Also, inhibition of IRE1α resulted in reduced expression of NLRP1 and neuronal pyroptosis associated proteins, demonstrating the important role of IRE1α and ER-stress in NLRP-1-induced neuronal pyroptosis, and the pathway by which NETs exert their impact post TBI (8).

*ER-stress and cell death*

There is a clear link between NETs, ER stress, and ER-stress mediated apoptosis. Similar to other studies, upon administration of NET modulators NET formation, neuronal ER stress and ER stress-mediated apoptosis in neurones was reduced (11). In this study, Toll-like receptor-9 (TLR9) was identified as a mechanism by which ER-stress and neuronal apoptosis is induced following formation of NETs, and one whereby suppression of NETs and the TLR9-ER stress signalling pathway resulted in improved outcomes following TBI. A critical role of TLR9 in initiating downstream signalling pathways mediating immune responses through pattern recognition receptors was discussed in the paper. TLR9 was shown to mediate NET-induced ER-stress activation and apoptosis, with TLR9 being shown to be primarily expressed in neurones in the peri-contused regions of the brain following TBI. Moreover, upon inhibition of TLR9, neuronal ER-stress and ER-stress induced neuronal apoptosis were both ameliorated following TBI. Overexpression of PAD4 was shown to aggravate neuronal ER-stress and ER-stress induced apoptosis following TBI, while also reducing anti-apoptotic markers. The impact of PAD4 (and NET) overexpression following TBI was reversed by administration of TLR9 antagonist, a finding that was validated in vitro. Together this demonstrates how NETs may contribute to neuronal damage post TBI through induction of ER-stress and ER-stress induced neuronal apoptosis, and that TLR9 has an important function in NET-induced neuronal ER-stress and apoptosis following TBI (11).

***Supplementary Discussion***

***Mechanistic outcomes***

Secondary brain injury following TBI is a complicated interlinked disease process, and it is established that neuroinflammation plays a key role in this pathological response (18). Reduced mortality in critically injured patients due to improvements in early trauma care means there is a greater significance put upon understanding the inflammatory response following traumatic injury and managing this process (19). This review provides evidence, through presenting findings from mechanistic sub-studies, and elucidates a role for the contribution of NETs towards secondary brain injury and in the inflammatory response. However, an in-depth discussion into the mechanistic nuances of the pathways and process which NETs cause disease and which modulation attenuate deficits is both beyond the scope of this review and yet to be fully elucidated. Nevertheless, the review provides some mechanical insight into how NETs induce various TBI secondary injuries. NETs influencing the inflammatory environment towards a pro-inflammatory state as well as ER-stress induced apoptosis linked to NET formation are key themes which the review identifies.

As discussed within the review, it is clear that NETs influence the inflammatory state towards a pro-inflammatory profile, including M1 microglial polarisation and CD11b+ pericyte appearance, through NET component interactions. Activated pericytes mediate neuroinflammation under pathological conditions by neutrophil infiltration and BBB disruption (20). CD11b is a transmembrane protein found on macrophages and microglia (21) and NET-associated histones were shown to induce CD11+ brain pericyte appearance following TBI, forming and adopting a microglial phenotype after TBI (1). These CD11b+ pericytes led to BBB disruption and neutrophil infiltration following TBI, with dectin-1 in pericytes identified as being responsible for recognising NET histones resulting in the induction of CD11b+ pericyte expression. Mechanistically, PKC and c-Jun, an AP1 protein, activation by dectin-1 was identified as important pathway protein interactions in the expression of CD11b+ pericytes. However, the mechanistic detail underlying dectin-1 and NET histone binding remains unknown. Also, the ability of Histones to promote CD11b expression was lower in comparison to NET medium, exhibiting that there are other potential components of NETs which contribute towards CD11b expression, which need to be elucidated.

Microglia play an essential role in the brains response to trauma and participate in deficits through proliferation and release of proinflammatory mediators and neurotoxic molecules, exacerbating TBI pathogenesis (22). Microglial activation by NETs and pro-inflammatory polarisation is a key feature described in multiple publications within the review and is recognised as a mechanism by which NETs cause disease, releasing subsequent detrimental proinflammatory mediators, with microglial mediated neuroinflammation and sympathetic hyperactivity ensuing deficits identified. The release of these detrimental proinflammatory mediator creates an unfavorable tissue microenvironment, resulting in neuronal dysfunction, chronic inflammation, and oxidative stress (23,24). NET mediated microglial activation plays an important pathological role following TBI. Zhu and colleagues identified how the NET mediated LL37-Hippo/MST1 signaling pathway induces microglial activation, through NETs interacting with microglial P2x7, secretion of neuroinflammatory cytokines, and sympathetic hyperactivity (10). However, the molecular mechanisms linking microglial activation and sympathetic hyperactivity remain to be defined, with Zhu and colleagues failing to evidence this. Qu and colleagues identified the HMGB1/JNK/AP1 signaling pathway in NET mediated microglial activation and verified a reduction in sympathetic hyperactivity when NETs are reduced. However, a molecular mechanism linking sympathetic hyperactivity and microglial activation following TBI was not defined (9).

STING, a transmembrane ER-protein, interacts with NET components and plays an important role in neuroinflammation and neuronal cell death after TBI (25). Upon NET component interactions, STING-dependent ER stress mediated deficits, including increased M1 microglia activation, decreased M2 microglia, and increased neuronal apoptosis and pyroptosis occur, alongside reduced functional outcomes. As reported by Shi and colleagues, JNK and AP1 were again common pathway proteins identified to be involved with NET mediated deficits, specifically ER-stress/IRE1α microglial activation, microglial mediated neuroinflammation ,and neuronal cell death (4). The appearance of JNK and AP1 proteins cemented the importance of these proteins across a range of NET mediated deficits. Additional in vitro experiments, as well as knockout (PAD4-/- and STING-/-) animal models, are required to validate NET mediated STING dependent deficits. Also, clarification of which NET components interact with STING following TBI to induce TBI-secondary injury defects is required, as well as elucidating the way in which they interact.

In terms of pathways, NET modulation appears to be neuroprotective by reversing the same pathways whereby NETs cause disease, leading to a reduction in TBI secondary injury deficits. Reductions in deleterious pathway proteins and secondary deficits are apparent upon NET modulation, with neuroprotective effects of NET modulation also being abolished upon increasing pathway sensitivities and upregulating pathway protein expression. It is also important to note, however, that in all the mechanistic experimental studies included in this review, markers of pathways proteins and secondary injury deficits were not restored to uninjured levels, suggesting involvement of other mechanisms and additional pathways. Studies have suggested that other components of NETs yet to be identified may also contribute to TBI secondary injury deficits through the pathways described and through alternative pathways. There is therefore a requirement to further elucidate the mechanisms by which NETs cause disease and how NET modulation results in reduction of TBI secondary injury deficits. Exploration into the mechanism by which NETs cause TBI secondary injury will allow for greater treatment efficacy due to improved understanding of pathophysiological mechanisms of secondary injury and treatment approaches.

***Supplementary conclusion***

In addition to functional outcomes, NET modulation was demonstrated to improve various TBI-associated pathophysiological deficits, providing further support for NETs as a potential therapeutic target. Also, the various mechanism whereby NETs exert effect and are responsible for the pathogenesis of various secondary TBI injuries have been alluded to. However, additional studies are required to elucidate the full molecular underpinnings of these mechanism, which will allow for greater treatment efficacy.

**List of abbreviations:**

AP1, activator protein 1; ASK1, apoptosis signal-regulating kinase 1; BBB, blood brain barrier; CBF, cerebral blood flow; cf-DNA, cell-free DNA; cGAMP, cyclic guanosine monophosphate-adenosine monophosphate; cGAS, cyclic guanosine monophosphate-adenosine monophosphate synthase; Cit-H3, citrullinated histone H3; DNase, deoxyribonuclease; ER-stress, endoplasmic reticulum-stress; HMGB1, high mobility group box 1; ICP, intracranial pressure; IRE1-α, inositol-requiring transmembrane kinase/endoribonuclease 1 alpha; LL37, human cathelicidin LL-37; MAPK, mitogen-activated protein kinase; mNSS, modified neurological severity score; MPO, myeloperoxidase; MST, mammalian sterile-twenty-like kinase; NE, neutrophil elastase; NET, neutrophil extracellular trap; NLRP1, nucleotide-binding oligomerization domain (NOD)-like receptor pyrin domain containing 1; P2x7, purinergic receptor P2x7; (p)-c-FOS, (phosphorylated)-proto-oncogene c-FOS; (p)-c-JUN, (phosphorylated)-Jun proto-oncogene; (p)JNK, (phosphorylated) Jun N-terminal kinase; PKC, protein kinase; PVN, paraventricular nucleus; STING, stimulator of interferon response cGAMP interactor 1; TBI, traumatic brain injury; TLR9, toll-like receptor 9

**Supplementary References:**

1. Liu YW, Zhang J, Bi W, Zhou M, Li J, Xiong T, et al. Histones of Neutrophil Extracellular Traps Induce CD11b Expression in Brain Pericytes Via Dectin-1 after Traumatic Brain Injury. Neurosci Bull. 2022 Oct 11;38(10):1199–214.

2. Vaibhav K, Braun M, Alverson K, Khodadadi H, Kutiyanawalla A, Ward A, et al. Neutrophil extracellular traps exacerbate neurological deficits after traumatic brain injury. Sci Adv. 2020 May 29;6(22).

3. Jin J, Wang F, Tian J, Zhao X, Dong J, Wang N, et al. Neutrophil extracellular traps contribute to coagulopathy after traumatic brain injury. JCI Insight. 2023 Mar 22;8(6).

4. Shi G, Liu L, Cao Y, Ma G, Zhu Y, Xu J, et al. Inhibition of neutrophil extracellular trap formation ameliorates neuroinflammation and neuronal apoptosis via STING-dependent IRE1α/ASK1/JNK signaling pathway in mice with traumatic brain injury. J Neuroinflammation. 2023 Sep 30;20(1):222.

5. Li B, Xu L, Wang Z, Shi Q, Cui Y, Fan W, et al. Neutrophil Extracellular Traps Regulate Surgical Brain Injury by Activating the cGAS-STING Pathway. Cell Mol Neurobiol. 2024 Dec 18;44(1):36.

6. Mu Q, Yao K, Syeda MZ, Wan J, Cheng Q, You Z, et al. Neutrophil Targeting Platform Reduces Neutrophil Extracellular Traps for Improved Traumatic Brain Injury and Stroke Theranostics. Advanced Science. 2024 Jun 23;11(21).

7. Gu Z, Li L, Li Q, Tan H, Zou Z, Chen X, et al. Polydatin alleviates severe traumatic brain injury induced acute lung injury by inhibiting S100B mediated NETs formation. Int Immunopharmacol. 2021 Sep;98:107699.

8. Cao Y, Shi M, Liu L, Zuo Y, Jia H, Min X, et al. Inhibition of neutrophil extracellular trap formation attenuates NLRP1-dependent neuronal pyroptosis via STING/IRE1α pathway after traumatic brain injury in mice. Front Immunol. 2023 Apr 14;14.

9. Qu X, Hou X, Zhu K, Chen W, Chen K, Sang X, et al. Neutrophil extracellular traps facilitate sympathetic hyperactivity by polarizing microglia toward <scp>M1</scp> phenotype after traumatic brain injury. The FASEB Journal. 2023 Sep 3;37(9).

10. Zhu K, Zhu Y, Hou X, Chen W, Qu X, Zhang Y, et al. NETs Lead to Sympathetic Hyperactivity After Traumatic Brain Injury Through the LL37-Hippo/MST1 Pathway. Front Neurosci. 2021 Apr 29;15.

11. Mi L, Min X, Shi M, liu L, Zhang Y, Zhu Y, et al. Neutrophil extracellular traps aggravate neuronal endoplasmic reticulum stress and apoptosis via TLR9 after traumatic brain injury. Cell Death Dis. 2023 Jun 26;14(6):374.

12. Wang Y, Hu H, Yin J, Shi Y, Tan J, Zheng L, et al. TLR4 participates in sympathetic hyperactivity Post-MI in the PVN by regulating NF-κB pathway and ROS production. Redox Biol. 2019 Jun;24:101186.

13. Wang Y, Yin J, Wang C, Hu H, Li X, Xue M, et al. Microglial Mincle receptor in the <scp>PVN</scp> contributes to sympathetic hyperactivity in acute myocardial infarction rat. J Cell Mol Med. 2019 Jan 24;23(1):112–25.

14. Yan X, Li F, Maixner DW, Yadav R, Gao M, Ali MW, et al. Interleukin‐1beta released by microglia initiates the enhanced glutamatergic activity in the spinal dorsal horn during paclitaxel‐associated acute pain syndrome. Glia. 2019 Mar 21;67(3):482–97.

15. Qi J, Zhao XF, Yu XJ, Yi QY, Shi XL, Tan H, et al. Targeting Interleukin-1 beta to Suppress Sympathoexcitation in Hypothalamic Paraventricular Nucleus in Dahl Salt-Sensitive Hypertensive Rats. Cardiovasc Toxicol. 2016 Jul 25;16(3):298–306.

16. Liu Q, Wang T, Yu H, Liu B, Jia R. Interaction Between Interleukin-1 Beta and Angiotensin II Receptor 1 in Hypothalamic Paraventricular Nucleus Contributes to Progression of Heart Failure. Journal of Interferon & Cytokine Research. 2014 Nov 5;34(11).

17. Liu MM, Rivera-Chavez F, Wolf S, Minei J, Gatson J. (Conference Abstract) Neutrophil extracellular traps (NETs) formation after traumatic brain injury. Crit Care Med [Internet]. 2012 Dec [cited 2024 Jun 28];40:1–328. Available from: https://journals.lww.com/ccmjournal/abstract/2012/12001/201__neutrophil_extracellular_traps__nets_.168.aspx

18. Nizamutdinov D, Shapiro L. Overview of Traumatic Brain Injury: An Immunological Context. Brain Sci. 2017 Jan 23;7(1):11.

19. Rogers E, Pothugunta S, Kosmider V, Stokes N, Bonomini L, Briggs GD, et al. The Diagnostic, Therapeutic and Prognostic Relevance of Neutrophil Extracellular Traps in Polytrauma. Biomolecules. 2023 Nov 7;13(11):1625.

20. Rustenhoven J, Jansson D, Smyth LC, Dragunow M. Brain Pericytes As Mediators of Neuroinflammation. Trends Pharmacol Sci. 2017 Mar;38(3):291–304.

21. Özen I, Deierborg T, Miharada K, Padel T, Englund E, Genové G, et al. Brain pericytes acquire a microglial phenotype after stroke. Acta Neuropathol. 2014 Sep 22;128(3):381–96.

22. Jassam YN, Izzy S, Whalen M, McGavern DB, El Khoury J. Neuroimmunology of Traumatic Brain Injury: Time for a Paradigm Shift. Neuron. 2017 Sep;95(6):1246–65.

23. Loane DJ, Kumar A. Microglia in the TBI brain: The good, the bad, and the dysregulated. Exp Neurol. 2016 Jan;275:316–27.

24. Hu X, Leak RK, Shi Y, Suenaga J, Gao Y, Zheng P, et al. Microglial and macrophage polarization—new prospects for brain repair. Nat Rev Neurol. 2015 Jan 11;11(1):56–64.

25. Zhang L, Xin Y, Wu Z, Song R, Miao H, Zheng W, et al. <scp>STING</scp> mediates neuroinflammatory response by activating <scp>NLRP3</scp> ‐related pyroptosis in severe traumatic brain injury. J Neurochem. 2022 Sep 2;162(5):444–62.

**Supplementary Table 1. Summary of supplementary results and other findings**

| Study ID | Summary of alternative functional outcomes | Summary of neurological and pathophysiological TBI-associated deficit outcomes | Summary of outcomes regarding reduction in NETs upon modulation | Summary of mechanistic findings |
| --- | --- | --- | --- | --- |
| Cao et al., 2023 | N/A | NET modulation ameliorated:  neuroinflammation, neuronal cell death, neuronal damage and necrosis, ER-stress, sympathetic hyperactivity | N/A | Correlation between NETs and ICP observed in humans following TBI  Following TBI, release of cf-DNA activates cascade series which activates STING protein; STING increases in neurones and microglia post TBI; STING correlates with NET formation and reduced upon NET modulation indicating STING participation in regulation of NET mediated deficits post TBI; NETs exert neurodestructive effects through inducing NLRP1 inflammasome-dependant neuronal pyroptosis through a STING- IRE1α-NLRP1 pathway |
| Gu et al., 2021 | N/A | NET modulation ameliorated: cerebral oedema, neuronal degeneration, inflammatory markers in lungs | Reduction in MPO, NE and Cit-H3 observed in the lungs | N/A |
| Jin et al., 2023 | NET modulation resulted in reduced 3-day mortality | NET modulation ameliorated: cerebral oedema, BBB dysfunction, coagulopathy | Reduction in Cit-H3 observed in the plasma | N/A |
| Li et al., 2024 | NET modulation resulted in improved neurological function (modified Garcia score) at 3- and 7-days | NET modulation ameliorated: cerebral oedema, neuroinflammation, neuronal cell death | Reduction in Cit-H3 and MPO-DNA observed in the plasma; reduction in Cit-H3 observed in the brain | Following TBI, release of cf-DNA activates cascade series which activates STING protein; STING increases in neurones and microglia post TBI; STING correlates with NET formation and reduced upon NET modulation indicating STING participation in regulation of NET mediated deficits post TBI; Inhibition of upstream STING activator protein alleviated brain injury induced deficits; Pre-treatment with cGAMP reversed effects of NET inhibition |
| Liu et al., 2022 | NET modulation resulted in improvements in anxiety, motor function and coordination, and survival rate | NET modulation ameliorated: BBB dysfunction | N/A | Following TBI histones from NETs induce CD11b+ pericyte appearance via interactions with dectin-1 in a PKC c-JUN dependant manner resulting in BBB disruption and neutrophil infiltration; other possible NET components that contribute to CB11b+ expression need to be elucidated |
| Mi et al., 2023 | N/A | NET modulation ameliorated: neuronal cell death, neuronal damage and necrosis, ER-stress | Reduction in Cit-H3 observed in the brain; failed to show significant reduction in MPO observed in the brain in two separate analyses | NET formation, neuronal ER-stress and ER-stress mediated apoptosis in neurones reduced upon NET modulation; TLR9 mediate NET induced ER-stress activation and apoptosis with TLR9 primarily expressed in neurones in peri-contused regions; inhibition of TLR9 alleviated neuronal ER-stress and ER-stress induced neuronal apoptosis |
| Mu et al., 2024 | N/A | NET modulation ameliorated: cerebral oedema, BBB dysfunction, neuroinflammation, neuronal cell death | Reduction in Cit-H3 and MPO observed in the brain | N/A |
| Qu et al., 2023 | N/A | NET modulation ameliorated: neuroinflammation and sympathetic hyperactivity | N/A | HMGB1 (NET component) contributes towards M1 phenotypic conversion via MAPK (p-JNK/JNK)/AP1(p-c-JUN/c-JUN and p-c-FOS/c-FOS) pathway resulting in sympathetic hyperactivity following TBI; Correlations between HMGB1, NET formation, microglial inflammatory factors and sympathetic hyperactivity observed following TBI; Anti-HMGB1 and AP-1 inhibitors reduced sympathetic excitability |
| Shi et al., | NET modulation resulted in improvements in forelimb motor function and postural and sensorimotor asymmetries | NET modulation ameliorated cerebral oedema, CBF, BBB dysfunction, neuroinflammation, neuronal cell death, degenerating neurones, neuronal damage and necrosis, ER-stress, sympathetic hyperactivity | Reduction in Cit-H3 and MPO observed in the brain | Following TBI release of cf-DNA activates cascade series which activates STING protein; STING increases in neurones and microglia post TBI; STING correlates with NET formation and reduced upon NET modulation indicating STING participation in regulation of NET mediated deficits post TBI; Inhibition of upstream STING activator protein alleviated brain injury induced deficits; Pre-treatment with cGAMP reversed effects of NET inhibition; NET formation orchestrated by PAD4 exert neurodestructive effects following TBI through STING-mediated IRE1α activation in microglia and neurones resulting in neuronal cell death and microglial induced neuroinflammation via the STING-dependant IRE1α/ASK1/JNK pathway; NET modulation results in reduced neuronal damage through regulation of IRE1α/ASK1/JNK pathway |
| Vaibhav et al., 2020 | NET modulation resulted in improvements in anxiety, motor function and coordination, motor coordination, neuromuscular function, and recognition memory | NET modulation ameliorated cerebral oedema, CBF | Reduction in MPO-DNA and cellular expression of MPO and NE on LgG6+TLR4+ neutrophils observed in the plasma; reduction in Cit-H3 and MPO expression on Ly6G+ neutrophils observed in the brain | Correlation between NETs and ICP observed in humans following TBI; NETs are mechanically distinct mediators of cerebral oedema formation and hypoperfusion demonstrated by improvements in deficits observed in subjects with diminished capacity to generate NETs; NET localisation observed in parallel with reduced CBF, persistent hypoperfusion, tissue hypoxia and reduced brain tissue oxygenation |
| Zhu et al., 2021 | N/A | NET modulation ameliorated: neuroinflammation, sympathetic hyperactivity | N/A | LL37 (NET component) interacts with P2x7 in microglia to promote the secretion of IL-1β via the Hippo/MST pathway. This, alongside correlation between NETs and sympathetic hyperactivity, suggests that NETs pose as distinct mediators of sympathetic hyperactivity induced through release of this cytokine |

AP1 = activator protein 1; ASK1 = apoptosis signal-regulating kinase 1; BBB = blood brain barrier; CBF = cerebral blood flow; Cf-DNA = cell-free DNA; cGAMP = cyclic guanosine monophosphate-adenosine monophosphate; Cit-H3 = citrullinated histone H3; ER-stress = endoplasmic reticulum stress; HMGB1 = high mobility group box 1; ICP = intracranial pressure; IRE1-α = inositol-requiring transmembrane kinase/endoribonuclease 1 alpha; LL37 = human cathelicidin LL-37; MAPK = mitogen-activated protein kinase; MPO = myeloperoxidase; MST = mammalian sterile-twenty-like kinase; NE = neutrophil elastase; NET = neutrophil extracellular trap; NLRP1 = nucleotide-binding oligomerization domain (NOD)-like receptor pyrin domain containing 1; P2x7 = purinergic receptor P2x7; (p)-c-FOS = (phosphorylated)-proto-oncogene c-FOS; p-c-JUN= phosphorylated-Jun proto-oncogene; (p)JNK = (phosphorylated) Jun N-terminal kinase; PKC = protein kinase; STING = stimulator of interferon response cGAMP interactor 1; TBI = traumatic brain injury; TLR9 = toll-like receptor 9
